# Lhx1 functions together with Otx2, Foxa2, and Ldb1 to govern anterior mesendoderm, node, and midline development

**DOI:** 10.1101/gad.268979.115

**Published:** 2015-10-15

**Authors:** Ita Costello, Sonja Nowotschin, Xin Sun, Arne W. Mould, Anna-Katerina Hadjantonakis, Elizabeth K. Bikoff, Elizabeth J. Robertson

**Affiliations:** 1 The Sir William Dunn School of Pathology, University of Oxford, Oxford OX1 3RE, United Kingdom;; 2 Developmental Biology Program, Sloan Kettering Institute, New York, New York 10065, USA

**Keywords:** Lhx1, definitive endoderm, mesendoderm, midline, node, Ldb1

## Abstract

Gene regulatory networks controlling functional activities of spatially and temporally distinct endodermal cell populations in the early mouse embryo remain ill defined. The T-box transcription factor Eomes, acting downstream from Nodal/Smad signals, directly activates the LIM domain homeobox transcription factor *Lhx1* in the visceral endoderm. Here we demonstrate Smad4/Eomes-dependent *Lhx1* expression in the epiblast marks the entire definitive endoderm lineage, the anterior mesendoderm, and midline progenitors. Conditional inactivation of *Lhx1* disrupts anterior definitive endoderm development and impedes node and midline morphogenesis in part due to severe disturbances in visceral endoderm displacement. Transcriptional profiling and ChIP-seq (chromatin immunoprecipitation [ChIP] followed by high-throughput sequencing) experiments identified Lhx1 target genes, including numerous anterior definitive endoderm markers and components of the Wnt signaling pathway. Interestingly, Lhx1-binding sites were enriched at enhancers, including the *Nodal*-proximal epiblast enhancer element and enhancer regions controlling *Otx2* and *Foxa2* expression. Moreover, in proteomic experiments, we characterized a complex comprised of Lhx1, Otx2, and Foxa2 as well as the chromatin-looping protein Ldb1. These partnerships cooperatively regulate development of the anterior mesendoderm, node, and midline cell populations responsible for establishment of the left–right body axis and head formation.

## Abstract (precis)

Costello et al. demonstrate that Smad4/Eomes-dependent *Lhx1* expression in the epiblast marks the entire definitive endoderm lineage, the anterior mesendoderm, and midline progenitors. In proteomic experiments, they characterize a complex comprised of Lhx1, Otx2, and Foxa2 as well as the chromatin-looping protein Ldb1.

Shortly after implantation, Nodal and Wnt signaling pathways coordinately instruct the symmetrical cup-shaped epiblast, the founder tissue of the embryo proper, to become appropriately patterned and give rise to the three primary germ layers: the ectoderm, endoderm, and mesoderm (Tam and Loebel 2007; Arnold and Robertson 2009). The anterior–posterior axis first becomes evident at the onset of gastrulation, when cells on the prospective posterior side of the epiblast undergo an epithelial-to-mesenchymal transition to form nascent mesoderm in the primitive streak (PS). Nodal and Wnt antagonists expressed in the anterior visceral endoderm (AVE) ensure that the anterior epiblast gives rise to neuroectoderm progenitors (Arnold and Robertson 2009; Fossat et al. 2012). The definitive endoderm (DE) progenitors, ingressing through the anterior PS (APS), initially intermingle with mesoderm, subsequently become polarized, and emerge onto the outer surface of the embryo, dispersing the visceral endoderm (VE) cells (Kwon et al. 2008; Viotti et al. 2014). Extension of the PS toward the distal tip of the epiblast leads to the formation of the anterior mesendoderm (AME), a specialized subset of cells that condense at the midline and displace the overlying VE (Yamanaka et al. 2007). A few hours later, a transient and architecturally distinct structure, the node, arises from the APS progenitors. By a process of convergent extension, the node gives rise to the notochord, which, together with the AME, forms a continuous midline cell population (Yamanaka et al. 2007), the source of key growth factor signals necessary to promote growth and patterning of the overlying neuroectoderm. Asymmetric Nodal signaling from the node specifies the left–right (L–R) body axis (Collignon et al. 1996), whereas the specialized midline cell population provides an essential barrier function to confine Nodal signaling to the left side of the embryo (Lee and Anderson 2008).

The T-box transcription factor (TF) *Eomesodermin* (*Eomes*) has been identified as a key regulator acting downstream from dose-dependent Nodal/Smad signals (Arnold et al. 2008). During gastrulation, *Eomes* controls allocation of cardiovascular and DE progenitors in the PS (Costello et al. 2011; Teo et al. 2011). Additionally, *Eomes* is required at earlier stages for specification and maintenance of the AVE (Nowotschin et al. 2013). Recent experiments demonstrate in the VE that *Eomes* directly activates the homeobox LIM domain TF *Lhx1* (*Lim1*) (Nowotschin et al. 2013). *Lhx1* is also transiently expressed in nascent mesoderm (Barnes et al. 1994; Shawlot and Behringer 1995; Perea-Gomez et al. 1999). Loss-of-function *Lhx1* mutant embryos display a severe block to gastrulation, characterized by constriction of the VE at the extraembryonic ectoderm/epiblast boundary that acutely disrupts morphogenetic cell movements (Shawlot and Behringer 1995). Decreasing *Lhx1* expression in the epiblast causes defective AME formation and consequently leads to anterior truncations (Shawlot et al. 1999; Fossat et al. 2015).

Here we demonstrate that Nodal/Smad signals activate *Eomes*-dependent *Lhx1* expression in the epiblast. As for the *Eomes*^+^ cell population (Costello et al. 2011), *Lhx1*^+^ progenitors exclusively colonize the head and cardiac mesoderm and the entire gut endoderm as well as APS derivatives, including the AME, node, and notochord. Consistent with previous studies (Fossat et al. 2015), we found here that conditional deletion from the epiblast does not perturb early mesoderm induction. However, high-resolution imaging revealed striking defects in anterior DE (ADE) emergence and dispersal of the VE population. Additionally, we demonstrated that epiblast-specific deletion profoundly disturbs node morphogenesis as well as formation of the embryonic anterior midline population.

To learn more about *Lhx1* functional contributions, we performed transcriptional profiling experiments. We identified Lhx1 targets, including numerous AME and DE marker genes, as well as components of the Wnt signaling pathway required for correct head patterning (Arkell et al. 2013). To gain further mechanistic insights, we also carried out a proteomic screen in stably transfected P19CL6 cells expressing epitope-tagged Lhx1 constructs. These results demonstrate Lhx1 interacts with its well-described binding partners, Ldb1 and Ssbp3 (Agulnick et al. 1996; Nishioka et al. 2005; Enkhmandakh et al. 2006). Additionally, we characterized a tripartite TF complex comprised of Lhx1, the forkhead family member Foxa2 (Hnf3β), and the paired-like homeobox protein Otx2. Finally, genome-wide chromatin immunoprecipitation (ChIP) followed by high-throughput sequencing (ChIP-seq) experiments demonstrate Lhx1 occupancy primarily at putative enhancer elements. Strikingly, Lhx1 binds to enhancer regions at both *Otx2* and *Foxa2*. Collectively, these findings strongly argue that Lhx1 functions together with its transcriptional partners, Otx2 and Foxa2, to coordinately regulate AME, node, and midline development.

## Results

### *Lhx1* expressed downstream from Smad/Eomes marks the DE lineage and midline progenitors

**Figure 1.**
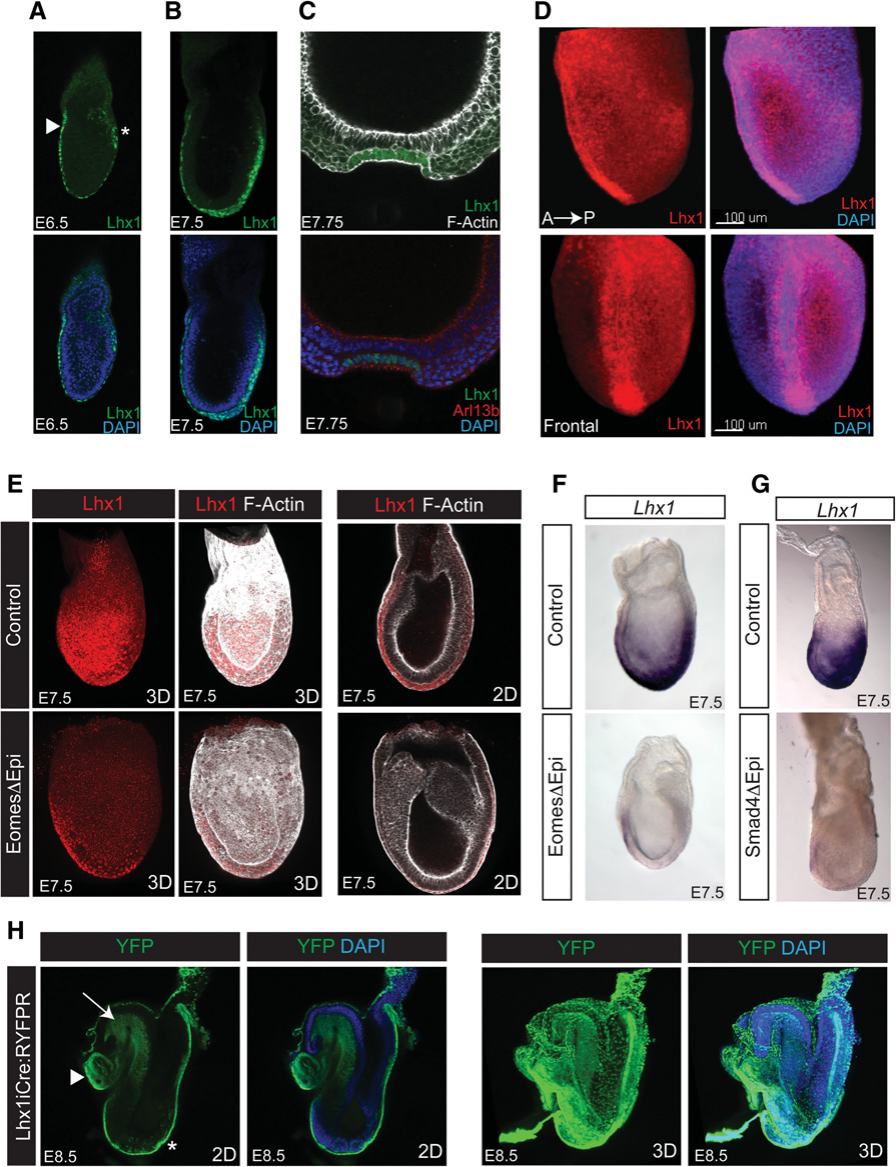
Smad/Eomes functional activities are required for Lhx1 expression in the epiblast. (*A*) Confocal microscopy reveals Lhx1 expression (green) at the onset of gastrulation in nascent mesoderm, emerging at the tip of the PS (asterisk) and the overlying VE and AVE (arrowhead). (*B*) Slightly later, at E7.5, Lhx1 expression marks nascent mesoderm, emerging along the PS and APS progenitors at the distal tip. (*C*) Arl13b-positive (red) ciliated cells localized within the ventral notochordal plate strongly express Lhx1. (*D*) Three-dimensional (3D) images at EHF stages reveal the highest levels of Lhx1 expression (red) within the midline mesoderm and node. (*E*) Eomes mutant (EomesΔEpi) embryos selectively lack epiblast expression (red), but Lhx1 expression is retained in the overlying VE. (*F*,*G*) Transient expression is lost in the early mesoderm and midline of EomesΔEpi (*F*) and Smad4Δepi (*G*) mutant embryos, but *Lhx1* expression is retained in the genetically wild-type AVE. (*H*) Two-dimensional (2D) and 3D imaging of E8.5 Lhx1^iCre^:ROSA26^RYFP^ reporter embryos reveals that Lhx1^+^ progenitors selectively give rise to the cranial mesoderm (arrow), heart mesoderm (arrowhead), and midline and DE progenitors (asterisk).

Beginning at embryonic day 6.5 (E6.5), Lhx1 is expressed throughout the VE overlying the epiblast (Fig. 1A) and a small number of mesoderm cells at the proximal rim of the posterior epiblast. A few hours later, Lhx1 is detectable in mesoderm, ingressing along the length of the PS as well as in the distal tip cells and the anterior midline mesendoderm (Fig. 1B). Subsequent to node formation, the ciliated ventral cells of the notochordal plate express high levels of Lhx1 (Fig. 1C). At the early headfold (EHF) stage, robust Lhx1 expression becomes confined to the node and midline, with lower levels detectable in the cranial and cardiac mesoderm (Fig. 1D).

Recent experiments demonstrated that the T-box TF Eomes directly activates *Lhx1* expression in the VE (Nowotschin et al. 2013). To test whether Eomes also acts upstream of *Lhx1* in the epiblast, we examined *Lhx1* expression in embryos carrying an epiblast-specific *Eomes* deletion (EomesΔEpi) (Arnold et al. 2008). The genetically wild-type VE retains *Lhx1* expression. However, conditional inactivation of *Eomes* eliminates *Lhx1* expression throughout the epiblast (Fig. 1E,F). *Smad4* function in the epiblast is known to be essential for specification of the APS, midline, and DE (Chu et al. 2004). *Smad4* conditional inactivation in the epiblast similarly results in failure to activate *Lhx1* expression in the epiblast (Fig. 1G).

Next, to trace the fate of Lhx1^+^ cells, we engineered a dual-purpose *Lhx1iCreIRESLacZ* reporter allele carrying *LacZ* and *Cre* expression cassettes introduced under the control of endogenous *Lhx1* regulatory elements (Supplemental Fig. S1). The *LacZ* reporter is transiently expressed at E6.5 in the AVE and nascent mesoderm and slightly later in the ventral node, AME, and midline. At early somite stages, a second domain of *Lhx1.LacZ* expression was detectable in the lateral nephrogenic mesoderm. To further characterize Lhx1^+^ derivatives, *Lhx1*^*iCreIRESLacZ*/+^ males were mated to females carrying either the *Rosa26*^*RLacZ*^ or *Rosa26*^*RYFP*^ reporter allele (Soriano 1999; Srinivas et al. 2001). As for Eomes^+^ epiblast cells (Costello et al. 2011), we also found that Lhx1^+^ LacZ progeny give rise to the head mesenchyme, heart, gut endoderm, node, and notochord (Supplemental Fig. S1H–J). To globally visualize YFP^+^ Lhx1 descendants, we used confocal microscopy and three-dimensional (3D) rendering software (Fig. 1H). Transient *Lhx1* expression labels the entire DE lineage. The rostro–caudal axis of the forming gut tube, from the most anterior foregut pocket to the hindgut diverticulum, is exclusively derived from Lhx1^+^ progenitors (Fig. 1H; Supplemental Fig. S1H–J).

### Conditional loss of *Lhx1* disrupts ADE and AME development

Partial loss (∼70%) of *Lhx1* from the epiblast causes abnormalities in anterior patterning associated with Wnt signaling defects (Tanaka et al. 2010; Fossat et al. 2015). To completely eliminate *Lhx1* function in the epiblast, we exploited the Sox2Cre deleter strain (Hayashi et al. 2002) together with a novel *Lhx1* conditional allele generated using a EUCOMM (European Conditional Mouse Mutagenesis Program) resource targeting vector (Supplemental Fig. S2). The resulting *Lhx1*^Δ/−^*:Sox2Cre*^*Tg*/+^ (hereafter referred to as Lhx1ΔEpi) mutant embryos (Supplemental Fig. S2F) appear morphologically normal at early stages but, by E9.5, display marked cardiac defects, including abnormal looping, expanded pericardium and cardia bifida, and a marked reduction of anterior endoderm and neural tissue (Supplemental Fig. S2G–I). By E10.5, all Lhx1ΔEpi mutants are growth-retarded and necrotic (Supplemental Fig. S2J).

**Figure 2.**
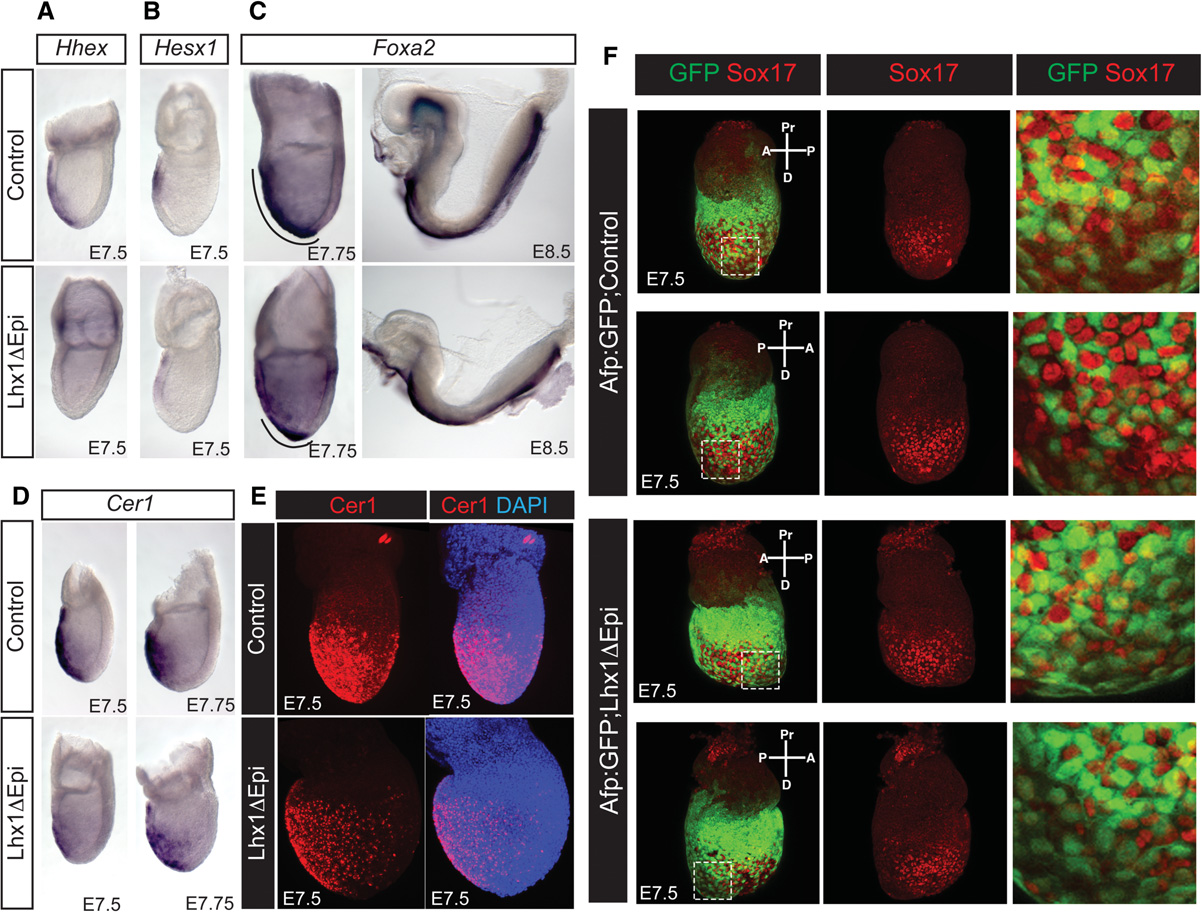
Conditional Lhx1 inactivation in the epiblast disrupts ADE specification and VE dispersal. (*A*,*B*) Expression of the ADE markers *Hhex* (*A*) and *Hesx1* (*B*) is barely detectable in Lhx1ΔEpi embryos. (*C*) Development of *Foxa2*-positive midline cells is severely compromised at both E7.75 and E8.5. (*D*,*E*) Cer1 mRNA (*D*) and protein (*E*) expression levels are markedly reduced in *Lhx1* mutant embryos. (*E*) Confocal imaging shows significantly fewer Cer1-positive (red) DE progenitors present on the ventral surface. (*F*) Confocal imaging of *Afp:GFP* (green) transgene expression and Sox17 (red) staining reveals dramatically reduced Sox17^high^ DE emergence and excessive representation of GFP^+^ VE cells at the distal tip of Lhx1ΔEpi embryos.

We observed in Lhx1ΔEpi mutants that expression of the ADE markers *Hhex* and *Hesx1* mRNA is severely compromised (Fig. 2A,B). *Foxa2* is normally expressed in the anterior midline mesendoderm, the developing node, and their derivatives (Fig. 2C; Sasaki and Hogan 1993). Lhx1ΔEpi mutants display markedly reduced *Foxa2* expression in the AME and emerging midline. By somite stages, Lhx1ΔEpi embryos lack *Foxa2* expression in the anterior midline underlying the forebrain/midbrain (Fig. 2C). However, node progenitors at the tip of the PS at E7.75 retain robust *Foxa2* expression (Fig. 2C). The Nodal/Bmp/Wnt antagonist *Cer1*, transiently expressed in the nascent DE emerging onto the surface of the embryo, is correctly induced in Lhx1ΔEpi mutant embryos, but, as assessed by whole-mount in situ hybridization (WISH) and confocal imaging of immunofluorescence data, the number of Cer1^+^ cells is significantly reduced (Fig. 2D,E). *Afp* expression transiently labels the VE overlying the epiblast, whereas the DE that emerges onto the surface epithelium lacks *Afp* expression. Importantly, as judged by WISH analysis, patchy *Afp* expression is retained in Lhx1ΔEpi mutants (Supplemental Fig. S3A), indicative of a disturbance in DE intercalation or delay of *Afp* down-regulation.

To further investigate *Lhx1*’s contributions to DE emergence, we exploited the well-characterized *Afp:GFP* transgenic reporter strain (Kwon et al. 2008) in combination with the pan-endodermal marker Sox17. Prior to E7.75, Sox17 is weakly expressed in the VE, whereas emerging epiblast-derived DE cells robustly express Sox17 (Sox17^high^). At late bud (LB)-EHF stages in wild-type embryos, the lateral posterior surface is comprised predominantly of Sox17^high^ DE cells, corresponding to the earliest ADE progenitors exiting the PS (Fig. 2F). In contrast, in Lhx1ΔEpi mutants, the representation of Sox17^high^GFP^−^ DE cells is greatly diminished, and a substantial proportion of the lateral surface remains covered by Sox17^low^GFP^+^ VE cells (Fig. 2F). Thus, we conclude that *Lhx1* is essential for specification of the initial population of APS progenitors giving rise to the AME and for efficient intercalation of the DE cell population into the overlying VE layer.

### Lhx1 plays a crucial role during midline and node morphogenesis

**Figure 3.**
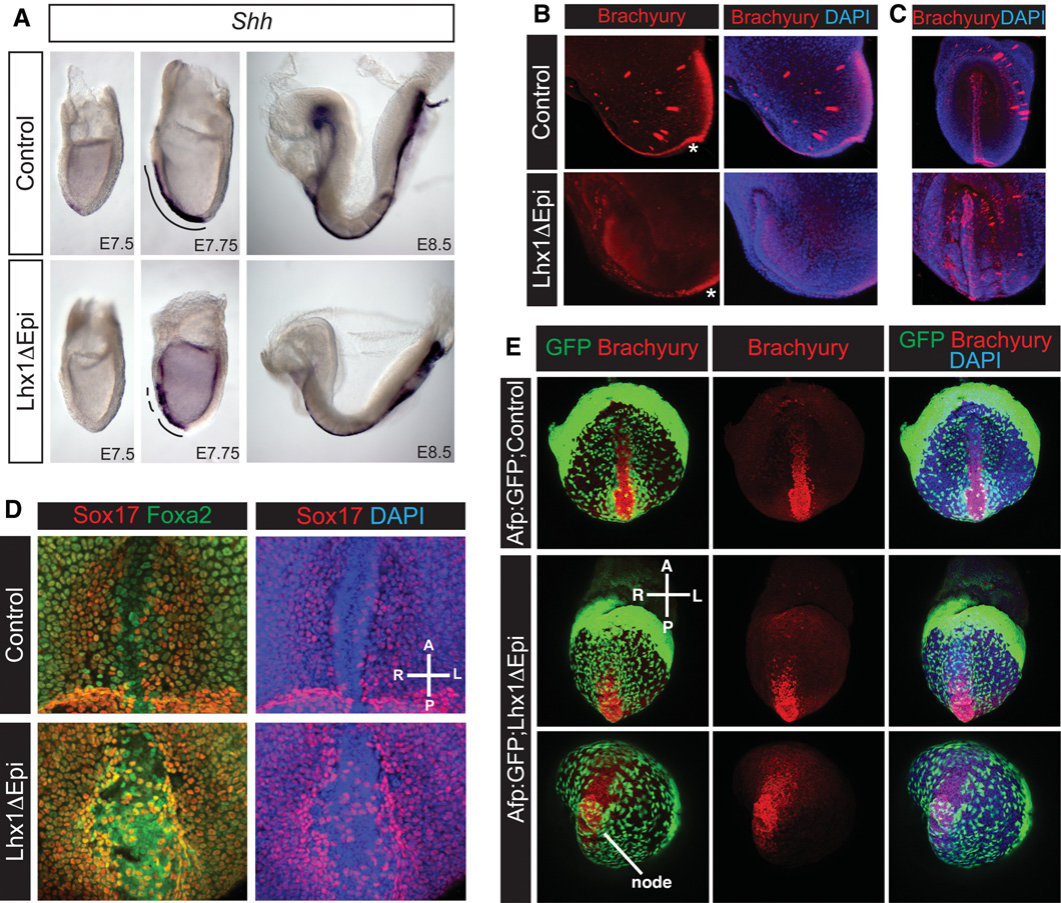
Lhx1 function is essential for midline morphogenesis. (*A*) *Shh* expression is absent at E7.5 in the node progenitors and subsequently, at E7.75, becomes discontinuous and fails to extend anteriorly. By E8.5, the anterior midline is severely disturbed. (*B*) At EHF stages, Brachyury is normally expressed in the PS, node (asterisk), and anterior midline, whereas, in Lhx1ΔEpi embryos, midline expression is patchy. The observed speckles are background staining. (*C*) Frontal view at slightly later stages (four- to five-somite stage) reveals discontinuous Brachyury staining in Lhx1ΔEpi mutants. (*D*) Sox17 (red) and Foxa2 (green) double staining of the emerging midline. In Lhx1ΔEpi embryos, midline Foxa2 staining is reduced, and the Sox17^+^ endoderm obscures the midline. (*E*) Analysis of *Afp:GFP* reporter expression in Lhx1ΔEpi embryos reveals defective node and midline development. The endoderm-obstructing node and midline emergence is GFP^+^ VE.

Sonic hedgehog (*Shh*) expressed in the anterior midline mesendoderm and posterior notochord precursors initiates dorsal–ventral patterning of the overlying neural plate (Chiang et al. 1996). In Lhx1ΔEpi embryos, early *Shh* expression is absent (Fig. 3A). By E7.75, expression along the anterior midline is discontinuous, and by E8.5, the anterior *Shh* domain that normally underlies the ventral forebrain is completely absent (Fig. 3A). Additionally, we observed patchy Brachyury expression in the anterior midline (Fig. 3B,C). Foxa2 expression in the emerging midline is also perturbed (Fig. 3D). Displacement of the overlying VE, an essential feature of midline development that normally allows the column of midline cells to emerge onto the ventral surface (Kwon et al. 2008; Viotti et al. 2012), is also severely compromised. Consistent with results above, when we evaluated Sox17 and *Afp:GFP* transgene expression in Lhx1ΔEpi mutant embryos, we observed that scattered Sox17^+^ and GFP^+^ VE cells partially conceal the compromised Foxa2^+^/Brachyury^+^ midline population (Fig. 3D,E).

**Figure 4.**
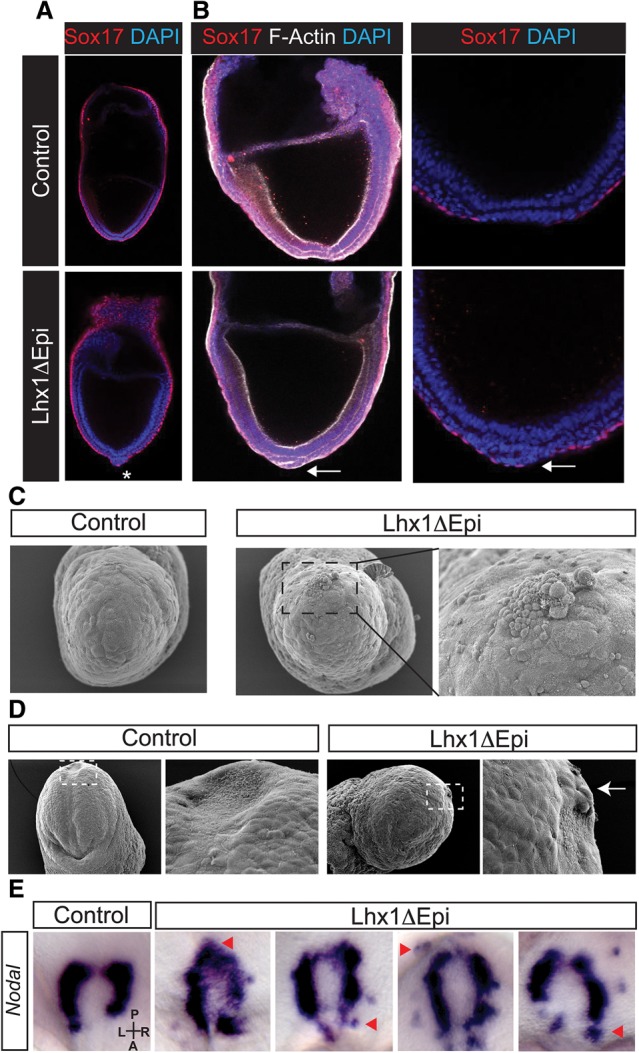
Lhx1 is required for node emergence and asymmetric Nodal expression. (*A*) The node is normally exposed at the distal tip of the embryo at LB stages. As judged by DAPI staining (blue), in Lhx1ΔEpi mutants, the node has condensed to form a bilayer structure but is obscured by Sox17^+^ endoderm (asterisk). (*B*) Slightly later, F-Actin staining (white) and DAPI nuclear staining (blue) demonstrate that the bilayered node has emerged. In contrast, in Lhx1ΔEpi-null embryos, the presumptive node remains encapsulated by Sox17^+^ endoderm cells (arrow). (*C*) SEM demonstrates a monolayer of endoderm overlying the distal tip, whereas, in contrast, cells accumulate on the surface of Lhx1ΔEpi embryos. (*D*) At EHF stages, the node is conspicuously visible as a concave, teardrop-shaped structure composed of monociliated cells. In contrast, in Lhx1Δepi embryos, squamous endoderm-like cells cover the posterior end of the node (arrow). (*E*) Unlike asymmetric left-sided *Nodal* expression in wild-type embryos, Lhx1ΔEpi embryos display bilateral, right-sided, and ectopic *Nodal* expression at the node (examples shown by arrowheads).

At the LB to EHF stages, the emerging node is normally exposed on the outer surface of the embryo. In contrast, a high proportion of Lhx1ΔEpi mutant embryos display a noticeable morphological thickening at the distal tip. Moreover, a continuous layer of Sox17^+^ endoderm cells conceals the ventral node (Fig. 4A,B). Scanning electron microscopy (SEM) confirmed in wild-type embryos, prior to the emergence of the node, an uninterrupted layer of endoderm (Fig. 4C), whereas Lhx1ΔEpi mutants are characterized by a local out-pocketing of cells on the surface of the distal tip (Fig. 4C). Slightly later, at EHF stages, when the patent bilayered node (consisting of ciliated cells on the ventral surface) normally becomes visible (Fig. 4D), we observed in Lhx1ΔEpi embryos that GFP^+^ VE cells obscure the posterior aspect of the node (Figs. 3E, 4D).

At early somite stages, asymmetric *Nodal* expression in the node selectively promotes its own induction in left lateral plate mesoderm (LPM) via an autoregulatory feed-forward loop that establishes the L–R body axis (Adachi et al. 1999; Norris and Robertson 1999). In Lhx1ΔEpi mutants, *Nodal* is ectopically expressed around the posterior end of the node and beyond its normal boundary (*n* = 5/5) (Fig. 4E). A similar ectopic expression is seen in *Sox17* mutants (Viotti et al. 2012). Despite robust expression in the node, asymmetric *Nodal* expression is disturbed and fails to be induced in the LPM of mutant embryos (*n* = 5/5). Considering that the DE plays an essential role in relaying signaling cues from the node to the LPM (Viotti et al. 2012), failure to activate *Nodal* in the LPM is probably caused by defective DE emergence.

### Transcriptional profiling identifies Lhx1 downstream target genes

**Figure 5.**
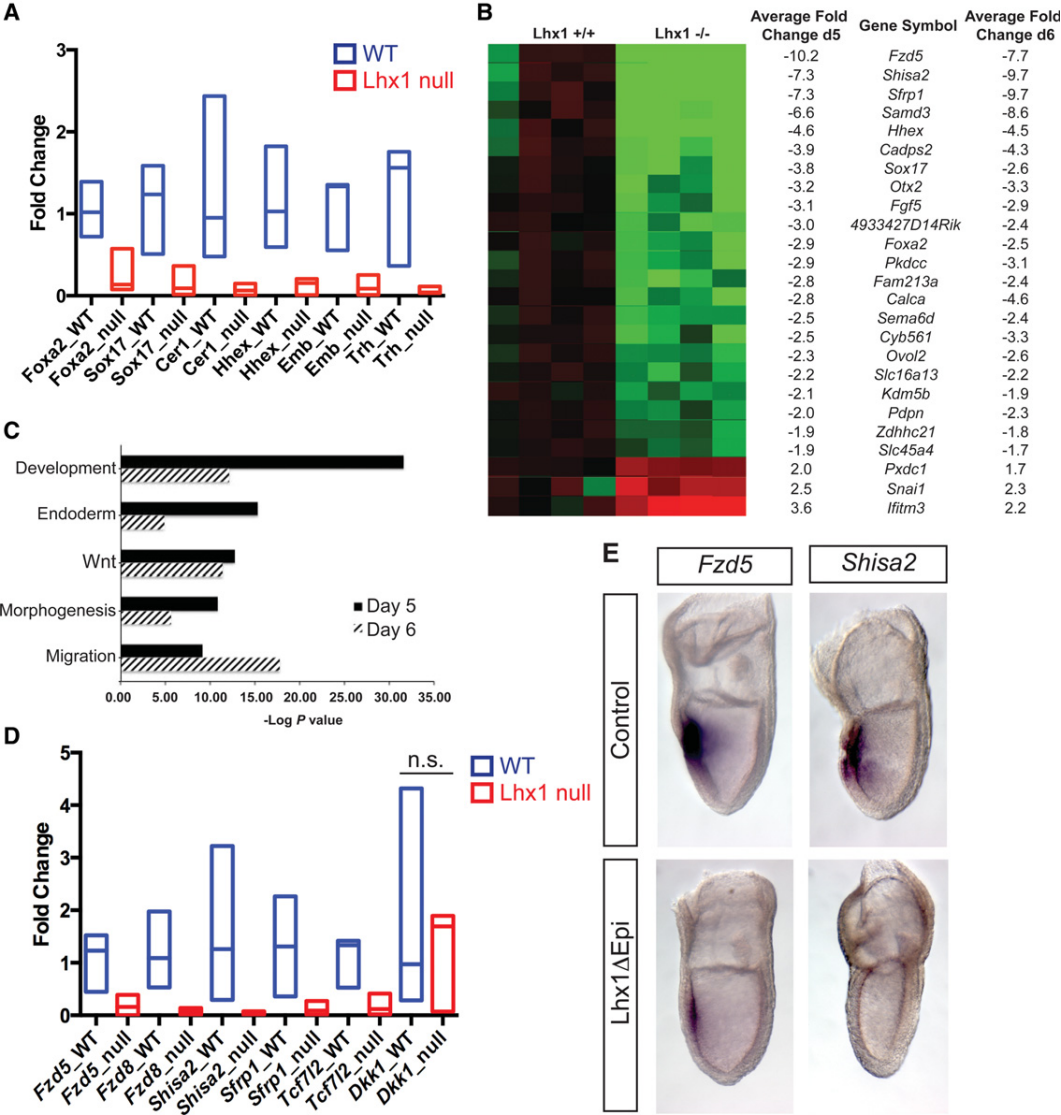
Transcriptional profiling experiments identify candidate Lhx1 targets. (*A*) Quantitative RT–PCR (qRT–PCR) analysis of endoderm and midline markers in day 6 ActivinA-treated embryoid bodies. (Blue bars) Wild-type samples (*n* = 4); (red bars) *Lhx1*-null samples (*n* = 4). Statistical analysis was performed using the Prism6 statistic package and the Student's *t*-test. Statistical significance, *P* < 0.05. The analysis is displayed as floating bars (minimum to maximum) with a line at the median. (*B*) Summary of genes misregulated at both day 5 and day 6 time points during embryoid body differentiation. The heat map indicates reduced (green) or increased (red) transcripts at day 5 as well as the average fold change at both day 5 and day 6. (*C*) Gene ontology (GO) of biological annotation identified high enrichment scores for the categories indicated. (*D*) qRT–PCR confirmed decreased expression of *Fzd5*, *Fzd8*, *Shisa2*, *Sfrp1*, and *Tcf7l2*, but *Dkk1* expression was not significantly (n.s.) altered at day 6. (Blue bars) Wild-type samples (*n* = 4); (red bars) *Lhx1*-null samples (*n* = 4). Statistical significance, *P* < 0.05. (*E*) WISH analysis shows markedly decreased *Fzd5* and *Shisa2* expression in the anterior midline.

To further characterize *Lhx1* functional contributions, we tested the ability of wild-type and *Lhx1*-null embryonic stem (ES) cells to differentiate toward APS fates in the presence of high doses of ActivinA (Morrison et al. 2008; Costello et al. 2011). As expected, mesodermal markers were efficiently induced (Supplemental Fig. 3B). The APS and DE markers *Gsc* and *Cxcr4* were also robustly expressed. In contrast, in the absence of *Lhx1*, expression of *Foxa2, Sox17*, and *Cer1* was significantly reduced (Fig. 5A). The ADE markers *Hhex* and *Hesx1* were barely detectable (Fig. 5A; Supplemental Fig. S3B). Interestingly, expression of *Embigin*, previously identified as a potential Lhx1 target in the AME (Shimono and Behringer 1999), and *Trh*, another DE marker (McKnight et al. 2007), are both dramatically reduced.

Next, we performed transcriptional profiling experiments. We identified 52 and 372 misregulated Ensembl annotated transcripts at day 5 and day 6, respectively (Illumina Diff Score >13, equivalent to *P* < 0.05) (Supplemental File 1). Of these, we focused on 25 transcripts misregulated at both time points: 22 down-regulated and three up-regulated (Fig. 5B). Functional annotation (gene ontology [GO]) clustering analysis of all misregulated genes with DAVID demonstrated high enrichment scores for development, endoderm, Wnt signaling, morphogenesis, and migration categories (Fig. 5C).

Consistent with the results above, DE and AME marker genes (namely, *Hhex*, *Sox17*, *Otx2*, and *Foxa2*) were markedly reduced. Additionally, several novel candidate Lhx1 target genes were identified: *Pkdcc* overlaps with *Lhx1* expression in the AVE, ADE, and AME (Imuta et al. 2009); *Calca* is coexpressed in the AME and node (Tamplin et al. 2011); and *Cyb561* is coexpressed in the node (Tamplin et al. 2008). Additionally, *4933427D14Rik*, *Sema6d*, *Kdm5b*, *Pdpn*, and *Ovol2* were coexpressed with *Lhx1* at the late streak (LS) to EHF stages (Supplemental Fig. S4B). Interestingly, *Shh*, *Col2a1*, *Epb4.1l5*, and *Shroom3*—genes previously shown to be required for proper midline, node, and neural morphogenesis (Chiang et al. 1996; Hildebrand and Soriano 1999; Lee et al. 2010; Leung et al. 2010)—were markedly down-regulated at day 6 of differentiation (−4.5-fold, −3.6-fold, −2.5-fold, and −2.1-fold, respectively) (Supplemental File 1). Overall, transcriptional profiling experiments confirm and extend the list of ADE, AME, and midline marker genes dependent on *Lhx1* expression.

Wnt signaling plays an essential role in patterning the anterior neuroectoderm (Arkell et al. 2013). GO analysis revealed that Wnt pathway components are significantly misregulated in Lhx1-null DE cultures (Fig. 5C). The Wnt receptors *Fzd5* and *Fzd8*, the Wnt antagonists *Shisa2* and *Sfrp1*, and the Wnt effector *Tcf7l2* are all significantly down-regulated (Fig. 5D; Supplemental Fig. S4C). A proposed Wnt target gene, *Apcdd1* (Takahashi et al. 2002), is up-regulated (Supplemental Fig. S4C). However, expression of *Wnt3*, *B-catenin*, *Frzb*, and *Sfrp2* as well *Dkk1*, an important Wnt antagonist essential for anterior patterning (Mukhopadhyay et al. 2001), was unaffected (Fig. 5D; Supplemental Fig. S4C). WISH experiments confirmed in Lhx1ΔEpi mutant embryos that expression of *Fzd5* and *Shisa2* is dramatically reduced (Fig. 5E). Therefore, Lhx1 modulates Wnt signaling by regulating the expression of multiple pathway components.

### Foxa2 and Otx2 interact with the core Lhx1 TF complex

**Figure 6.**
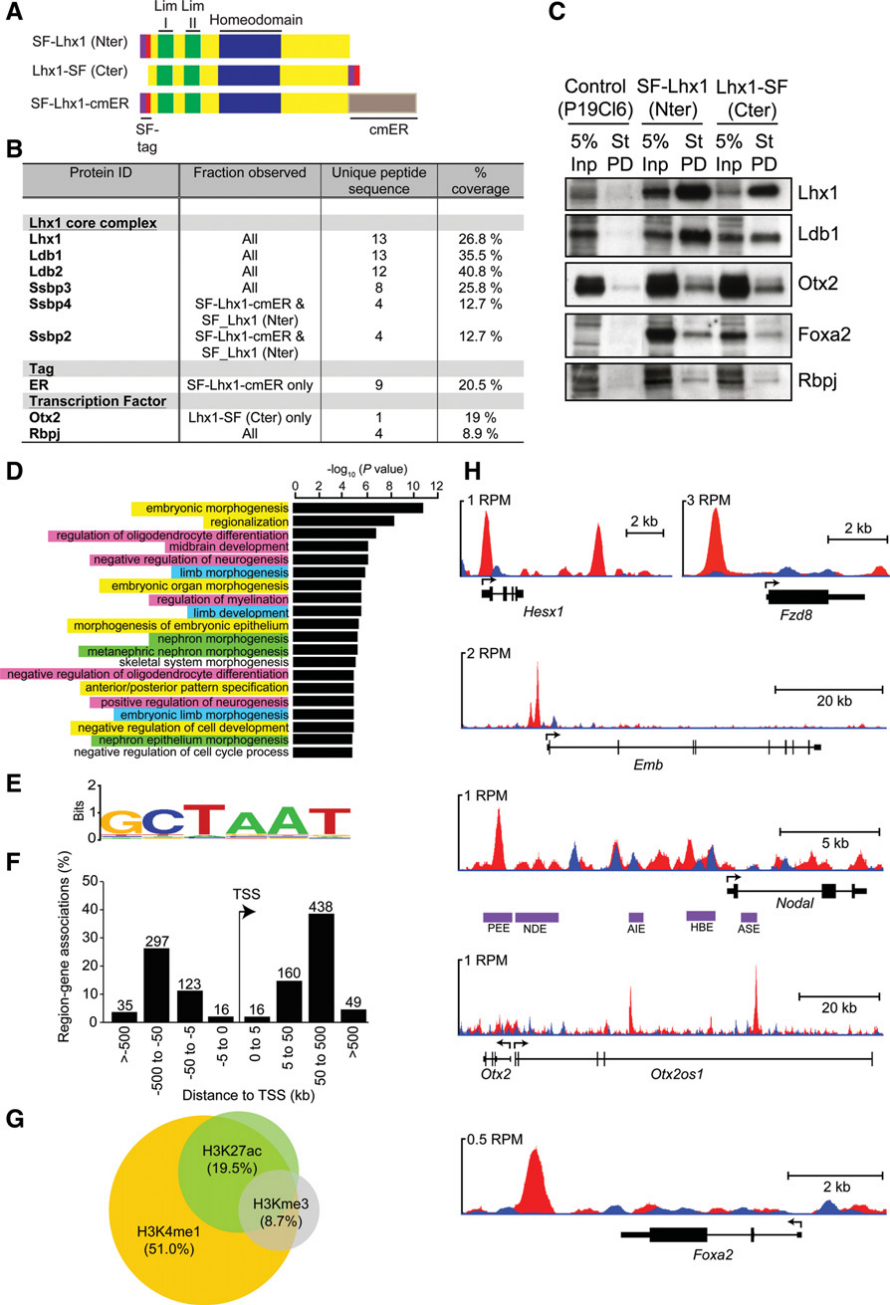
Identification of Lhx1 interaction partners and candidate target genes. (*A*) Schematic representation of SF epitope-tagged expression constructs. (*B*) Summary of select Lhx1 protein partnerships identified by MS analysis. The percentage of coverage for each protein, the number of unique peptide sequences, and corresponding pull-down fractions are shown. (*C*) Western blot analysis of StrepTactin pull-down (St-PD) experiments. As a positive control, 5% of the input (Inp) sample was analyzed. Proteins, indicated at the *right*, were enriched in test pull-down fractions (SF-Lhx1 Nter and Lhx1-SF Cter). (*D*) Functional annotation analysis using GREAT reveals that Lhx1 preferentially binds to genes associated with development, differentiation, and morphogenesis processes; namely, embryonic (yellow highlight), neural (pink), renal (green), and limb (blue) *Lhx1*-expressing tissues. (*E*) De novo motif analysis (Weeder) reveals an enrichment of a TAAT-containing sequence underlying Lhx1 peaks. (*F*) The distance from the nearest transcription start site (TSS) for each ChIP-seq peak and the number of peaks within each grouping are indicated. (*G*) Lhx1 ChIP-seq peaks were compared with previously reported histone modification profiles in mouse ES cells. (*H*) University of California at Santa Cruz (UCSC) track view of ChIP (red) and input (blue) wiggle plot overlays showing enrichment of Lhx1 ChIP-seq density at *Hesx1*, *Fzd8*, *Embigin*, *Nodal*, *Otx2*, and *Foxa2.* Purple boxes indicate the positions of previously mapped *Nodal* enhancer elements. (RPM) Reads per million.

DMSO-treated P19CL6 embryonal carcinoma cells transiently adopt a mesendodermal-like state, as judged by robust coexpression of *Lhx1, Eomes, T, Foxa2*, and *Cxcr4* (Costello et al. 2011). To learn more about Lhx1 functional activities, we performed an unbiased proteomic screen using expression constructs containing full-length *Lhx1* cDNA tagged with either an N-terminal (SF-Lhx1) or a C-terminal (Lhx1-SF) StrepFlag (SF) sequence (Fig. 6A). Additionally, to stimulate nuclear import in response to tamoxifen treatment, we added a C-terminal-ER sequence to the SF-Lhx1 N-terminal construct. Nuclear extracts from stably transfected differentiated (day 4) P19CL6 subclones were precipitated using StrepTactin resin and subjected to mass spectrometry (MS) analysis. A complete list of interacting proteins is presented in Supplemental File 2.

Lhx1 interacts with Ldb and Ssbp proteins to form an Lhx–Ldb–Ssbp core complex (Agulnick et al. 1996; Hobert and Westphal 2000; Nishioka et al. 2005; Enkhmandakh et al. 2006). The present experiments demonstrated in differentiating P19CL6 cells that Lhx1 binds to conserved components of the core complex (Fig. 6B; Supplemental File 2), including Ldb1 and Ldb2 as well as Ssbp2, Ssbp3 and Ssbp4. Interestingly, as for *Lhx1*, both *Ldb1* and *Ssbp3* mutant embryos similarly display anterior patterning defects (Mukhopadhyay et al. 2003; Nishioka et al. 2005; Enkhmandakh et al. 2006). These findings strongly suggest that Lhx1, Ldb1, and Ssbp3 cooperatively govern ADE specification and midline morphogenesis.

The homeobox TF Otx2 has been shown to bind Lhx1 and Foxa2 in vitro (Nakano et al. 2000). The present MS and Western blot analyses similarly demonstrate Lhx1 associations with Otx2 (Fig. 6B,C). Due to evidence for an interaction between Otx2 and Foxa2, we sought to identify whether Lhx1 could also interact with Foxa2. Lhx1–Foxa2 interactions were clearly detectable by StrepTactin pull-down and immunoblotting experiments (Fig. 6C). These results directly demonstrated for the first time in the context of mesendodermal cells a tripartite TF complex comprised of Lhx1, Foxa2, and Otx2.

The Notch signaling pathway plays an essential role in node morphogenesis (Przemeck et al. 2003; Raya et al. 2003). As for Lhx1ΔEpi mutants, embryos lacking *Rbpj* or the Notch ligand *Delta-1* (*Dll1*) display a spectrum of node and midline defects (Oka et al. 1995; Przemeck et al. 2003; Raya et al. 2003). In Lhx1ΔEpi mutants, as in *Dll1*-null embryos, the ventral surface of the node remains covered with endoderm, preventing its emergence (Przemeck et al. 2003). We found that Rbpj, a downstream effector of the Notch signaling, was present in all three test fractions (Fig. 6B). Western blot analysis confirmed Rbpj associations with Lhx1 complexes (Fig. 6C), suggesting that Lhx1 acts together with Rbpj during node emergence and L–R axis patterning.

### Lhx1 preferentially binds to putative enhancer elements governing target gene expression

To identify Lhx1 transcriptional targets genome-wide, we performed ChIP-seq experiments using differentiated P19CL6 cells stably transfected with the C-terminal cmER-tagged SF expression construct (Supplemental File 3). GREAT analysis revealed embryonic morphogenesis, anterior/posterior pattern specification, and morphogenesis of embryonic epithelium as terms associated with Lhx1-binding events (Fig. 6D). De novo motif finding identified the TAAT-containing sequence within the Lhx1 peak regions (Fig. 6E), confirming that Lhx1 binds to the previously described TAAT core motif recognized by several homeodomain TF family members (Berger et al. 2008).

Analysis of the genomic distribution of binding sites relative to the nearest transcription start site (TSS) revealed peak enrichments 5–500 kb on either side of and not immediately proximal to (<5 kb) the TSS, suggesting that Lhx1 may preferentially bind to enhancer regions (Fig. 6F). To evaluate this possibility, we compared Lhx1 ChIP-seq peaks with histone modification profiles reported for mouse ES cells (Fig. 6G). Interestingly, the majority of our Lhx1 peaks overlap with H3K4me1 peaks (Fig. 6G), known to be enriched at enhancer regions, and 26.1% of ChIP-peak regions overlap p300-bound regions in ES cells (Supplemental Fig. S5A). A smaller proportion of bound regions also display H3K27ac in ES cells, a marker of active enhancers, while only 8.7% of bound sites contain H3K4me3 modifications, associated with promoter regions (Fig. 6G).

Several putative Lhx1 target genes were represented in our ChIP data set. For example, Lhx1 binding was detected at two distinct regions at the *Hesx1* locus, including a regulatory element in the 5′ untranslated region (containing two Lhx1-binding motifs) and a 3′ distal enhancer (Fig. 6H; Chou et al. 2006). Lhx1 ChIP peaks were also present upstream of *Embigin* exon 1 (Fig. 6H). *Embigin,* an IgG superfamily member normally expressed in the VE, ADE, and forebrain neuroepithelium (Shimono and Behringer 1999; Sousa-Nunes et al. 2003), is down-regulated in *Lhx1*-null embryos (Shimono and Behringer 1999) and, as reported here, differentiated DE cultures (Fig. 5A). Thus, *Embigin* represents a direct Lhx1 target. One of the strongest Lhx1 ChIP peaks lies upstream of the *Fzd8* gene (Fig. 6H). Moreover, *Fzd8* is down-regulated in *Lhx1* mutant DE cultures (Fig. 5D). Additionally, Lhx1 peaks were also found associated with *Sfrp1* and *Tcf7l2* (Supplemental File 3). We conclude that Lhx1 directly regulates components of the Wnt pathway.

The node-specific *Nodal* enhancer (NDE) contains Rbpj-binding sites governing Notch-dependent *Nodal* expression (Raya et al. 2003). However, there was no evidence for Lhx1 occupancy within the NDE (Fig. 6H), and, as shown above, *Nodal* induction in the node is Lhx1-independent. Rather, we found Lhx1 enrichment at the *Nodal* proximal epiblast enhancer (PEE) element responsible for controlling *Nodal* expression levels in the PS (Norris and Robertson 1999; Vincent et al. 2003). Interestingly, as for Lhx1ΔEpi embryos, Nodal^ΔPEE/−^ embryos display severe defects in the formation of the APS derivatives and develop anterior truncations (Vincent et al. 2003). Moreover, the Rbpj-binding motif TGGGAA (Castel et al. 2013) is present within the PEE sequence element (Supplemental Fig. S5B,C). These findings strongly argue that Lhx1/Rbpj interactions cooperatively fine-tune Nodal signaling in the PS to govern dose-dependent formation of APS progenitors.

Otx2 has been shown to bind 1.5 kb upstream of and 6.5 kb downstream from the *Lhx1* TSS (Ip et al. 2014). Reciprocally, the present experiments identified prominent Lhx1 ChIP-seq peaks upstream of *Otx2* within both the EP/AN1 (epiblast/anterior neuroectoderm1) and FM1 (forebrain/midbrain1) enhancer regions (Fig. 6H; Kurokawa et al. 2004a,b). Additionally, we detected Lhx1 binding 3′ to the *Foxa2* gene at a site partially overlapping with the floor plate enhancer (Fig. 6H; Sasaki and Hogan 1996), suggesting that Lhx1 directly regulates expression of its transcriptional partners, Foxa2 and Otx2. This feed-forward regulatory loop in turn is required for AME and midline development.

Collectively, our results demonstrate Lhx1 interactions with Foxa2 and Otx2. Consistent with this, examination of previously published Otx2 and Foxa2 ChIP data sets (from ActivinA-treated EpiLCs or ES-derived DE cells, respectively) (Xu et al. 2012; Buecker et al. 2014) revealed overlapping binding sites (Supplemental Fig. S6). Of the high-confidence Lhx1 peaks, 114 (18.4%) were directly overlapping with the Foxa2 sites. These observations strengthen our proposed model in which the Lhx1/Foxa2/Otx2 TF complex cooperatively regulates the same genomic regions.

## Discussion

It is well known that specification of APS derivatives requires highest levels of Nodal/Smad signaling (Vincent et al. 2003; Chu et al. 2004; Ben-Haim et al. 2006; Morsut et al. 2010). This instructive cue guides formation of midline, node, and DE progenitors, the key cell populations that promote growth and patterning of the neuroectoderm and establishment of the L–R body axis. The T-box TF Eomes acts downstream from Nodal to control allocation of mesodermal and DE progenitors in the APS (Arnold et al. 2008; Costello et al. 2011). The present experiments demonstrated that Eomes directly activates *Lhx1* expression in the epiblast. Moreover, ChIP-seq analysis revealed Lhx1 enrichment at the Tcf/Lef-dependent *Nodal-*proximal epiblast enhancer. These findings strongly suggest that Lhx1 acts synergistically with the Wnt signaling pathway to sustain the positive feedback loop that amplifies Nodal signaling in the PS necessary to specify APS progenitors (Ben-Haim et al. 2006).

We demonstrated for the first time that *Lhx1* plays a pivotal role in axial midline and node morphogenesis. Early EM and imaging studies provided descriptive insights into the timing and emergence of these architecturally distinct structures (Lee and Anderson 2008). Beginning at E7.5, the cells of the prospective ventral node as well as clusters of midline cells located more anteriorly coalesce, become columnar in shape, and gradually emerge on the embryos’ surface. Displacement of the overlying VE cells allows these columnar cells to expand and eventually occupy the entire midline ventral surface (Kwon et al. 2008). In Lhx1ΔEpi embryos, the genetically wild-type VE cells fail to displace appropriately and prevent the orderly emergence of the node and axial midline precursors.

Interestingly, our transcriptional profiling experiments identified several known regulators of cellular behavior—including *Epb4.1l5*, *Shroom3*, and *Col2a1*—that are markedly down-regulated. Loss of *Epb4.1l5* disrupts apical–basal polarity and leads to a disordered F-Actin cytoskeletal network (Lee et al. 2007). Node precursors induced in *Epb4.1l5* mutants fail to organize and displace the overlying VE, resulting in L–R axis defects (Lee et al. 2010). *Epb4.1l5* is required for apical accumulation of another important cytoskeletal regulator, *Shroom3* (Chu et al. 2013). Targeted loss of *Shroom3* function causes neural tube defects (Hildebrand and Soriano 1999). *Col2a1* encodes the extracellular matrix type II collagen proteins (procollagens IIA and IIB). Procollagen IIA mutants display decreased *Shh* expression in the midline and partially penetrant head truncation phenotypes (Leung et al. 2010). Our ChIP-seq analysis revealed proximal Lhx1 binding at the *Shroom3* locus. Thus, Lhx1 may directly regulate cellular architecture.

The present results confirm and extend earlier work suggesting that *Lhx1* modulates the strength of Wnt signaling in the midline to ensure correct anterior neuroectoderm patterning (Fossat et al. 2015). Our transcriptional profiling experiments demonstrated that expression of multiple Wnt signaling components depends on Lhx1 activity. *Fzd5* (a Wnt receptor) and *Shisa2* (a Wnt antagonist normally expressed in the AME) are significantly down-regulated. Importantly, ChIP-seq experiments identified several Wnt pathway genes—including *Fzd8*, *Sfrp1*, and *Tcf7l2*—as direct Lhx1 targets.

Recent evidence suggests that *Otx2*, coexpressed with *Lhx1* in the AME, regulates *Lhx1* expression levels (Ip et al. 2014). Likewise, *Lhx1* and *Foxa2* are coexpressed in APS progenitors, the node, and the midline. *Foxa2*-null embryos fail to form a node and lack axial mesoderm-derived structures (Ang and Rossant 1994). *Foxa2* is critical for polarization of DE cells and epithelization of the midline structures (Burtscher and Lickert 2009). Conditional *Lhx1* ablation selectively within the *Foxa2* expression domain disrupts head development (Fossat et al. 2015). Our ChIP-seq experiments identified Lhx1-binding sites present at key enhancer regions controlling expression of *Otx2* and *Foxa2.* Additionally, protein interaction experiments clearly demonstrated Lhx1 interactions with both Otx2 and Foxa2. Intriguingly, *Lhx1*, *Otx2*, and *Foxa2* are also coexpressed in the embryonic VE at early post-implantation stages. All three loss-of-function alleles independently result in profound defects within the VE, causing a constriction at the interface of the proximal extraembryonic ectoderm and distal epiblast (Ang and Rossant 1994; Shawlot and Behringer 1995; Ang et al. 1996). Thus, the tripartite TF complex comprised of Lhx1/Foxa2/Otx2 probably activates common gene regulatory networks required in VE, DE, and AME lineages.

Additionally, we characterized for the first time a higher-order Lhx1/Ldb1/Ssbp3 complex assembled in mesendodermal cell cultures. Ssbp3 stabilizes the complex, regulating Lhx1 and Ldb1 steady-state levels (Gungor et al. 2007; Xu et al. 2007), thereby ensuring proper complex stoichiometry. *Ldb1* mutant embryos arrest during early development and display severe patterning defects, including constriction at the extraembryonic/embryonic boundary and anterior truncations (Mukhopadhyay et al. 2003). Ldb1 has been described as a looping factor that mediates long-range promoter enhancer interactions (Deng et al. 2012; Krivega et al. 2014). During erythroid cell differentiation, Ldb1 complexes with lineage-restricted TFs Gata1, Tal1, and Klf1 to activate β-globin gene expression (Love et al. 2014). Recently, in cardiac progenitors, Ldb1 has been shown to complex with another homedomain TF, Isl1, to regulate *Mef2c* and *Hand2* transcription (Caputo et al. 2015). The fact that defects caused by targeted disruption of the *Lhx1* LIM domains (responsible for Ldb1 interaction) also phenocopy those seen in *Lhx1*-null embryos (Cheah et al. 2000) strongly argues that associations with Ldb1 are essential for Lhx1 function.

**Figure 7.**
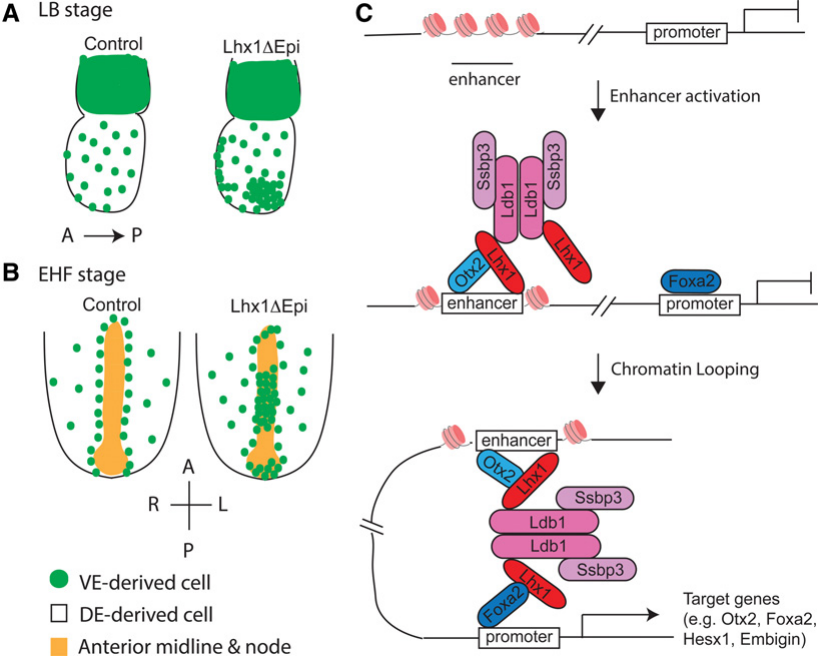
Summary of Lhx1 functional roles. (*A*) At LB stages, DE cells (white) normally migrate onto the outer surface, dispersing the VE (green) cells. In Lhx1ΔEpi mutants, the overlying VE is not properly dispersed, leaving pockets enriched with VE-derived cells. (*B*) At the EHF stage, the midline and node have emerged to form a continuous layer with the endoderm. In Lhx1ΔEpi mutants, VE-derived cells remain overlying the midline and posterior node. (*C*) Hypothetical model showing Lhx1 DNA binding at enhancers together with Otx2 acting as a pioneer factor to recruit higher-order Lhx1–Ldb1–Ssbp3 complexes and promote Foxa2 binding to promoter regions and activate transcription of target genes.

Distal enhancers are brought into close proximity with promoter regions to activate developmentally regulated target gene expression. Otx2 and Foxa2 are enriched at enhancers and promoters, depending on the cell context (Wederell et al. 2008; Bochkis et al. 2012; Xu et al. 2012; Buecker et al. 2014; Yang et al. 2014). The simplest possibility (depicted in Fig. 7) is that Lhx1, via its interactions with Otx2 and Foxa2, selectively recruits the Ldb1 chromatin-looping machinery to coordinately regulate transcriptional programs required for ADE, node, and midline development.

## Materials and methods

### Animals and PCR genotyping

*Eomes^CA/N^;Sox2Cre* (Arnold et al. 2008), *ROSA26*^*RLacZ*^ (Soriano 1999), *ROSA26*^*RYFP*^ (Srinivas et al. 2001), *Lim1*^+/−^ (Shawlot and Behringer 1995), and *AFP:GFP*^*TG*/+^ (Kwon et al. 2006) strains were genotyped as described. A novel *Lhx1*^*CA*^ allele was generated using the EUCOMM targeting vector (PGRS0002_B_E08) from the German Research Center for Environmental Health. AsiSI-linearized vector (15 µg) was electroporated into CCE ES cells, and neomycin-resistant colonies were screened by Southern blot analysis using the restriction enzyme and the probe combination shown in Supplemental Figure. S2. The offspring derived from two independent correctly targeted clones were crossed with FLPe mice (Farley et al. 2000) to remove the LacZ and Neo cassettes. The resulting *Lhx1*^*CA/CA*^ strain was maintained on a mixed 129SvEv/C57BL/6 background. *Lim1*^+/−^;*Sox2-Cre*^*Tg*/+^ males were crossed with *Lhx1*^*CA/CA*^ females to generate Lhx1^Epiblast-deleted/−^ (Lhx1ΔEpi) embryos.

To generate the *Lhx1*^*iCre-LacZ*^ reporter allele, a codon-optimized *Cre-*IRES-nlacZ followed by a FRT-flanked *neo* cassette (iCre-IRES-nlacZ-FRT-neo-FRT) (Mould et al. 2012) was introduced in-frame into exon 1. Correctly targeted clones were identified by Southern blot analysis (Supplemental Fig. S1) and transiently transfected with a FLP expression construct to remove the neo cassette. PCR genotyping primers for *Lhx1*^*CA*/+^ *and Lhx*^*iCre*^ mice are shown in Supplemental Table S1. All animal procedures were approved by the Ethical Review Committee of the University of Oxford and the Institutional Animal Care and Use Committee of Memorial Sloan Kettering.

### ES cell culture

Wild-type control and *Lhx1*-null ES cell lines were derived from Lhx1Δ/+ intercross blastocysts in 2i/LIF medium: N2B27 medium (StemCells, Inc.) supplemented with 1 µM PD0325901, 3 µM CHIR99021, and LIF (Millipore). Established ES cell lines were maintained in DMEM (Invitrogen) with 15% FBS, 1% nonessential amino acids, 0.1 mM β-mercaptoethanol, and 1000 U/mL recombinant LIF (Millipore). To induce DE formation, ES cells were seeded at low density (5 × 10^3^ cells per milliliter) in the absence of LIF in bacteriological-grade plates, and 50 ng/mL ActivinA (R&D systems) and 20 ng/mL EGF (Peprotech) were added after 48 h in N2B27 medium (based on Morrison et al. 2008).

### Immunofluorescence

Embryos were fixed in 4% paraformaldehyde for 30 min at room temperature; permeabilized with 0.5% Triton-X in PBS for 15 min; washed with 0.1% Triton-X in PBS; blocked in 5% donkey serum, 0.2% BSA, and 0.1% Triton-X in PBS for 1 h at room temperature; incubated with primary antibodies overnight at 4°C; washed in 0.1% Triton-X-PBS; incubated with fluorophore-conjugated secondary antibodies (AlexaFluor, Invitrogen); and counterstained with DAPI and/or Alex fluor 633 phalloidin (Invitrogen). The antibodies used are listed in Supplemental Table S2. Laser-scanning confocal data were acquired on an Olympus FV1000 or Zeiss LSM880, and image data were processed using ImageJ, ZEN software, and Bitplane Imaris software.

### SEM

Embryos fixed in 2.5% glutaraldehyde for at least 24 h were post-fixed with osmium tetroxide, dehydrated with a graded alcohol series, and critical point-dried from liquid CO_2_. Specimens were mounted on aluminum stubs with double-sided tape and coated with gold and were viewed at 5 kV on a JEOL scanning electron microscope.

### RNA analysis

RNA was prepared and analyzed by one-step and quantitative RT–PCR (qRT–PCR) as described (Costello et al. 2011) using the primer sequences listed in Supplemental Table S3. For transcriptional profiling experiments, RNA was isolated from four independent *Lhx1*-null (biological replicates) and wild-type control ES cell lines induced to differentiate into DE at days 4, 5, and 6. cRNA was hybridized to Illumina Mouse WG-6 v2 Expression BeadChips as described previously (Harper et al. 2011). Differential probe expression was determined following rank invariant normalization by using the Illumina custom error model option with Benjamini and Hochberg false discovery rate. Probes with significant different expression (differential score >13, equivalent to *P* < 0.05) were analyzed by using DAVID Bioinformatics Resources 6.7 (http://david.abcc.ncifcrf.gov).

### In situ hybridization, X-gal staining, and histology

WISH and X-gal staining were performed as before (Costello et al. 2011). The antisense ribroprobes used are described in Supplemental Table S4. For histology, embryos were post-fixed in 4% paraformaldehyde, dehydrated in ethanol, embedded in paraffin wax, sectioned (8 μm), and eosin-counterstained.

### Generation of SF-tagged Lhx1-expressing P19CL6 embryonal carcinoma cell lines

To generate stably expressing SF-tagged Lhx1 sublines, linearized pCAG-SF-Lhx1-IRES-Puro (N-terminal), pCAG-Lhx1-SF-IRES-Puro (C-terminal), or pCAG-SF-Lhx1-cmER-IRES-Puro vectors were introduced by electroporation, and drug-resistant clones were selected in 1 μg/mL puromycin and screened by Western blot analysis. For SF-Lhx1-cmER activation, 1 μg/mL 4-hydroxytamoxifen (Sigma, H7904) was added to the culture medium. P19CL6 cells were induced to differentiate via DMSO addition as previously described (Costello et al. 2011).

### ChIP-seq analysis

For ChIP-seq analysis, two independent SF-Lhx1-cmER-expressing clones and control P19CL6 cells were induced to differentiate for 4 d in the presence of DMSO and tamoxifen. ChIP was performed as described (Costello et al. 2011) using anti-Lhx1 antibody (Santa Cruz Biotechnology, sc-19341x) or control goat IgG (Santa Cruz Biotechnology, sc-2088). Eluted DNA samples recovered using a “ChIP DNA Clean and Concentrator” column kit (Zymo Research) were multiplexed and sequenced using two lanes on an Illumina HiSeq 2000 sequencer. Sequence reads were mapped to the mm9 mouse genome release with Stampy using default parameters (Lunter and Goodson 2011). Peak calling was performed using MACS1.4.2 (Zhang et al. 2008) using default parameters to call areas of enrichment. De novo motif finding within ChIP-seq peaks was performed using Weeder version 1.4.2 (Pavesi et al. 2004). The distribution and functional annotation of Lhx1 ChIP-seq peaks were performed using GREAT version 2.0.2 using the basal plus extension rule, annotating genes within 5 kb of TSSs initially and within 1 Mb where no proximal genes exist (McLean et al. 2010). Terms with a binomial *P-*value of ≤1 × 10^−5^ were considered significant. For comparison, H3K4me1, H3K4me3, H3K27ac, and p300 ChIP-seq peak coordinates were downloaded from NCBI Gene Expression Omnibus (GEO) accession numbers GSE31039 and GSE36027, while Otx2 and Foxa2 were downloaded from NCBI GEO accession numbers GSM1355169 and GSM993787, respectively. Regions of overlap between the ChIP-seq peaks identified in the present study and other published data sets were compared using custom Perl scripts.

### StrepTactin pull-downs and MS analysis

StrepTactin pull-down experiments were performed using nuclear extracts from SF-Lhx1 (N-terminal), Lhx1-SF (C-terminal), or SF-Lhx1-cmER stably transfected or control P19Cl6 cells induced to differentiate for 4 d. Twenty micrograms of Avidin per milligram of extract was added to block endogenous biotin, and nuclear extracts were treated with 1.25 U/mg benzonase. For large-scale precipitation, 15 mg of nuclear extract was incubated with StrepTactin Superflow resin (Iba, 2-21206) for 2 h, and the bound fraction was recovered in elution buffer (100 mM Tris, 150 mM Nacl, 1mM EDTA, 2.5 mM DesthioBiotin, 10% glycerol, 0.5 mM DTT) and subjected to electrophoresis in a 4%–20% miniprotean TGX gel (Bio-Rad, 456-1094S). Gel-excised fragments were digested with trypsin and analyzed by MS. Data were acquired on a Thermo Q Exactive mass spectrometer coupled to a Dionex RSLC nano-high-performance liquid chromatography system. Raw data files were converted to .MGF file format and analyzed using the Central Proteomics Facility Pipeline (Trudgian et al. 2010), and label-free quantitation was performed using SINQ (Trudgian et al. 2011). Criteria for identifying specific interactions included protein identification, including 1% false discovery rate and one or more unique peptides representative of each full-length sequence. Proteins present in control P19CL6 samples were filtered out of the final interpretation.

### Accession numbers

The microarray and ChIP-seq data have been deposited in NCBI GEO with accession number GSE70958.

## Supplementary Material

Supplemental Material
