# Supplementary material for: Lhx1 functions together with Otx2, Foxa2, and Ldb1 to govern anterior mesendoderm, node, and midline development
Source: Genes Dev. 2015 Oct 15;29(20):2108–22. doi: 10.1101/gad.268979.115 (PMC4617976; doi:10.1101/gad.268979.115)
Supplement: Supplemental Material [file supp_29_20_2108__index.html]

Supplemental Material 

# Lhx1 functions together with Otx2, Foxa2, and Ldb1 to govern anterior mesendoderm, node, and midline development

## Supplemental Material

**Files in this Data Supplement:**

- Supp Material.pdf
- Supp File 1.xlsx
- Supp File 2.xlsx
- Supp File 3.xlsx
