# Supplementary material for: Lhx1 functions together with Otx2, Foxa2, and Ldb1 to govern anterior mesendoderm, node, and midline development
Source: Genes Dev. 2015 Oct 15;29(20):2108–22. doi: 10.1101/gad.268979.115 (PMC4617976; doi:10.1101/gad.268979.115)

SUPPLEMENTAL FIGURES

Supplemental Figure S1: Generation of a dual purpose Lhx1-iCre-IRES-LacZ allele

(A) Schematic representation of the targeting strategy used to create the Lhx1-iCre-nLacZ allele. The iCre and nuclear LacZ expression cassette was inserted into the first exon of *Lhx1* downstream of the endogenous ATG. Targeted clones were identified using 5' and 3' external probes (red lines). RV, EcoRV; S, SphI. (B) Southern blot analysis of EcoRV or SphI digests in combination with 5' probe or 3' probes, respectively, detected diagnostic wild-type or targeted fragments as indicated. (C) PCR genotyping to distinguish WT and Lhx1$^{iCrenLacZ/+}$ heterozygous (T) mice. (D) WISH analysis confirms *iCre* and *LacZ* expression faithfully mirrors that of endogenous *Lhx1*. (E) At E6.5 nLacZ expression is detectable in the AVE and migrating lateral mesoderm wings. Beginning at E7.0, DE is also LacZ positive. At E7.75, LacZ staining is observed in the node and midline, as well as in the foregut pocket. By E8.0 embryos, LacZ expression in confined to the lateral mesoderm. (F) Transverse section at E7.5 reveals LacZ staining in the AME and prechordal plate. (G) Sagittal section of LHF stage embryo shows LacZ expression in the node, anterior endoderm and head mesenchyme. (H) Fate mapping experiments demonstrate that descendants of Lhx1-iCre expressing cells in *Lhx1*$^{iCre\text{-}nLacZ/+}$ embryos contribute to the gut tube (Gt), head mesenchyme (Hm) and embryonic heart (He). (I) At E8.0 & E8.5 Lhx1 descendants are present in head mesenchyme, gut and heart. (J) By E9.5 Lhx1iCre expressing cells have colonized the heart, the foregut (asterisk) and hindgut (arrowhead).

Supplemental Figure S2: Generation of a novel Lhx1 conditional allele

(A) Strategy used to generate a Lhx1 conditional allele (CA). The Eucomm targeting vector contains LoxP sites flanking the exons 2-3 (red arrows) and a Frt-flanked

(purple arrows) cassette containing En2-IRES-LacZ-pA (LacZ) and a drug selectable marker, neoR (hβActP-neo-pA). RV, EcoRV; RI, EcoRI; S, SphI. (B) Recombination at the Frt sites removes the LacZ and neo cassette, to create the $Lhx1^{CA}$ allele. Cre-mediated deletion generates the *Lhx1* null allele. Diagnostic primer combinations are indicated by arrows. (C) SphI digested DNA was analyzed by Southern blot using the 3' probe or EcoRV digests in combination with the 5' probe and EcoRI digested DNA was analysed with a neo probe to confirm correctly targeted clones. (D) PCR analysis confirmed LoxP sites were intact. (E) Multiplex PCR genotyping screen using the indicated primers (B). (F) Immunofluorescence analysis of Lhx1ΔEpi embryos confirmed the selective loss of Lhx1 expression in the mesoderm and emerging DE. (G) At E9.5 Lhx1ΔEpi embryos lack head structures anterior of the otic vesicle (asterisk) and commonly have an enlarged or inappropriately looped heart or display cardia bifida. (H) WISH analysis at E8.5 using the forebrain and midbrain marker *Otx2* confirms absence of expression in Lhx1ΔEpi embryos. (I) Analysis of *Afp:GFP* reporter expression in Lhx1ΔEpi embryos and littermate control embryos (~20ss) reveals an increased proportion of GFP+ VE in the remnant of the anterior foregut (arrow). Green, GFP; blue, nuclei; red, F-Actin. (J) At E10.5, Lhx1ΔEpi embryos are highly abnormal and growth retarded. Mutant embryos that are recovered have pinheads, enlarged pericardial sacs, display cardia bifida and are highly necrotic.

Supplemental Figure S3: Loss of Lhx1 results in defective DE specification *in vitro* and *in vivo*

(A) WISH analysis of LS to EHF stage embryos. *Afp* expression is detectable in VE but not DE cells. In Lhx1ΔEpi mutants, *Afp* expressing VE cells fail to be dispersed or displaced. (B) Wild-type and *Lhx1* null (*Lhx1*-/-) ES cells were cultured in the presence of high doses of ActivinA. Semi-quantitative RT-PCR analysis reveals expression of the mesoderm markers *Brachyury* and *Lefty2* is unaffected, whereas

expression of DE genes is compromised (*Foxa2, Sox17* and *Cer1*) in *Lhx1*-null cultures. *Hesx1* and *Hhex* expression is also markedly reduced.

Supplemental Figure S4: WISH and RT-PCR analysis of mis-regulated transcripts

(A) WISH analysis demonstrates down-regulated *Otx2* expression in Lhx1ΔEpi embryos. (B) WISH analysis of mis-regulated genes identified in *Lhx1* null DE cultures. (C) Semi-quantitative RT-PCR analysis reveals mis-regulated expression of Wnt pathway components. Expression of Wnt receptor *Fzd8*, Wnt antagonists *Shisa2* and *Sfrp1*, as well as the downstream mediator *Tcf7l2* are reduced in *Lhx1* null cultures. Other components of the pathway, including Wnt antagonists *Sfrp2* and *Dkk1*, the Wnt ligand *Wnt3* and the core canonical Wnt pathway component β-catenin (*Ctnnb1*) are unaffected. Expression of the Wnt target gene *Apcdd1* is up-regulated.

Supplemental Figure S5: Lhx1 ChIP-seq peaks are associated with enhancer elements

(A) Percentage of Lhx1 ChIP-seq peaks identified here in comparison with previously reported histone modification profiles in mouse ES cells. (B) UCSC gene view of the *Nodal* locus. Purple boxes underneath indicate the positions of the previously identified *Nodal* enhancer elements. Asterisks indicate the position of known (black) or hypothetical (red) Rbpj motif. (C) *Nodal* 5' sequence showing the proximal enhancer in blue (PEE) and the node enhancer in red (NDE). The identified TGGGAA Rbpj sequence motifs are boxed.

Supplemental Figure S6: Overlap of Lhx1, Otx2 and Foxa2 ChIP-seq profiles

UCSC track view of *Hesx1, Fzd8*, *Embigin*, *Nodal, Otx2* and *Foxa2* (expansion of data shown in Figure 6H). Lhx1 ChIP-seq peaks (red) in differentiated P19Cl6 cells transfected with a tagged Lhx1 expression construct, compared with Otx2 ChIP-seq peak profiles (green) in ActivinA-treated EpiLC cells (GEO accession number GSM1355169) and Foxa2 peak regions (light blue) in ES-derived definitive endoderm cells (GEO accession number GSM993787, called peaks with P value < 1 x 10^{-5}). In examples shown, there is an overlap of Lhx1 peaks with Otx2 and/or Foxa2 bound regions. In some cases, there is additional Otx2 (e.g. *Fzd8*) or Foxa2 (e.g. *Otx2*) bound regions in the genomic locus, which may be important in the proposed looping function of the Lhx1-Ldb1 complex.

Supplemental Table S1: Genotyping primers

Gene name	Forward primer sequence	Reverse primer sequence	Product
SoxCre	GCATAACCAGTGAAACA GCATTGCTG	GGACATGTTCAGGGATC GCCAGGCG	280 bp
Lim1_Null (Lhx1-)	GCTCTGATGCCGCCGTG TTCC	CTTCGCCCAATAGCAGC CAGTCC	200 bp
Lhx1_WT	AATCTGCCAGGCTTTTGC G	CTCCGTAGAAATAAATAC CCCGC	187 bp
Lhx1_CA	AATCTGCCAGGCTTTTGC G	CTCCGTAGAAATAAATAC CCCGC	329 bp
Lhx1_Δ	AATCTGCCAGGCTTTTGC G	GGACCTTGGGCTTTTGAA CTG	270 bp
Lhx1_iCre	ACTCTAATTGCCCTGTCG CTCC	GCATTCTAGTTGTGGTTT GTCC	450 bp

Supplemental Table S2: Antibodies used in this study

**Name**	**Species**	**Catalog number**	**Company**
Arl13b	Mouse monoclonal	ab136648	Abcam
Brachyury	Goat polyclonal	Sc-17743	SantaCruz
Brachyury	Goat polyclonal	AF2085	R&D Systems
Cer1	Goat polyclonal	AF1986	R&D Systems
Foxa2	Rabbit polyclonal	ab40874	Abcam
GFP-488	Rabbit polyclonal	A21311	Invitrogen

Lhx1	Goat polyclonal	sc-19341	Santa Cruz
Sox17	Goat polyclonal	AF1924	R&D Systems
Ldb1	Goat polyclonal	sc-11198	Santa Cruz
Otx2	Rabbit polyclonal	ab21990	Abcam
Rbpj	Goat polyclonal	sc-8213	Santa Cruz

Supplemental Table S3: RT-PCR primers

Gene Name	Forward primer	Reverse primer	Product
Apcdd1	CTACCCAGAGGGTGTTCTAC	CAATGGTCAGGTCAGCCTTG	153 bp
Brachyury	AACTTTCCTCCATGTGCTGAGAC	TGACTTCCCAACACAAAAAGCT	533 bp
Cer1	GGAGGAAGCCAAGAGGTTCT	GTCTTCATGGGCAATGGTCT	145 bp
Ctnnb1	TGGGACTCTGCACAACCTTTCTCA	AGTGTCGTGATGGCGTAGAACAGT	132 bp
Cxcr4	GGCTGTAGAGCGAGTGTTGC	GTAGAGGTTGACAGTGTAGAT	390 bp
Dkk1	CTGAAGATGAGGAGTGCGGCTC	GGCTGTGGTCAGAGGGCATG	183 bp
Embigin	CTCTTGGTGGCCATCATTCT	TCAGTTTTTCTGTACCGGGG	152 bp
Eomes	TGTTTTCGTGGAAGTGGTTCTGGC	AGGTCTGAGTCTTGGAAGGTTCATTC	323 bp
Foxa2	TGGCTGCAGACACTTCCTACT	CAACATCAGTACAACCCCTCTGGT	487 bp
Foxa2	CCCGGGACTTAACTGTAACG	TTGCTCACGGAAGAGTAGCC	147 bp
Frzb	TGCAAATGTAAGCCTGTCAGAGC	TCCACAACGGCGGTCACATC	122 bp
Fzd5	GTCTGTGCTGTGCTTCATC	AGTGACACACACAGGTAGCA	123 bp
Fzd8	GTTCAGTCATCAAGCAGCAAGGAG	AAGGCAGGCGACAACGACG	122 bp
Gsc	GCACCATCTTCACCGATGAG	CGGCGGTTCTTAAACCAGAC	145 bp
Hesx1	ACAGACCCTGGACAGACACC	TTCGTCCTCGGTACCAACTC	204 bp
Hhex	GAGGTTCTCCAACGACCAGA	GTCCAACGCATCCTTTTTGT	202 bp
Hprt	GCTGGTGAAAAGGACCTCT	CACAGGACTAGAACACCTGC	249 bp
Lefty2	CAGCCAGAATTTTCGAGAGG	CCTAGAGTCGATGAGGGCAG	265 bp
Lhx1	GTACCAAATGCGCCGGTTGT	CCGGTGGAGAGCTGCTTGTT	124 bp
Sfrp1	ACGAGTTGAAGTCAGAGGCCATC	ACAGTCGGCACCGTTCTTCAG	197 bp
Sfrp2	ATCCTGGAGACAAAGAG	TGACCAGATACGGAGC	142 bp

	CAAGACC	GTTGATG	
Shisa2	GACAGCTCGGCAGTCCC CAT	GTCTCCATCAGGCGGTT GGC	167 bp
Sox17	TTTGTGTATAAGCCCGAG ATGG	AAGATTGAGAAAACACG CATGAC	448 bp
Sox17	CAGAACCCAGATCTGCA CAA	GCTTCTCTGCCAAGGTC AAC	79 bp
Tcf7l2	AGAGAGTGCAGCCATCA ACCAGAT	CTGCATGTGAAGCTGTC GTTCCTT	112 bp
Trh	CTGGAAGCAGCCCAGGA G	CCGGATGCTGGCGTTTT G	163 bp
Wnt3	CAAGCACAACAATGAAG CAGGC	TCGGGACTCACGGTGT TTCTC	199 bp

Supplemental Table S4: Summary of *in situ* probes

**Probe**	**Source/Reference**
4933427D14Rik	Image clone 6406803
Afp	Waldrip *et al*., (1998)
Cer1	Belo *et al*., (1997)
Hhex	Thomas *et al*., (1998)
Hesx1	Thomas & Beddington, (1996)
Kdm5b	Image clone 6826125
Foxa2	Sasaki & Hogan, (1996)
Fzd5	Image clone 5292250
Lhx1	Barnes *et al*., (1994)
Nodal	Collignon *et al*., (1996)
Otx2	Ang *et al*., (1994)
Ovol2	Image clone 4981833
Pdpn	Image clone 4985288
Sema6d	Image clone 6406252
Shh	Echelard *et al*., (1993)
Shisa2	Image clone 5068886

Ang SL, *et al*., (1994) “Positive and negative signals from mesoderm regulate the expression of mouse Otx2 in ectoderm explants”, Development, 120, 2979-2989

Belo JA, *et al*., (1997) “Cerberus-like is a secreted factor with neutralizing activity expressed in the anterior primitive endoderm of the mouse gastrula” Mechanisms of Development, 68, 45-57

Barnes JD, *et al*., (1994) “Embryonic expression of Lim-1, the mouse homolog of Xenopus Xlim-1, suggests a role in lateral mesoderm differentiation and neurogenesis”, Dev Biol, 161, 168-178

Collignon J, *et al*., (1996) “Relationship between asymmetric *nodal* expression and the direction of embryonic turning”, Nature, 381, 155-158

Echelard Y, *et al*., (1993) “Sonic hedgehog, a member of a family of putative signaling molecules, is implicated in the regulation of CNS polarity”, Cell, 75, 1417-1430

Sasaki H & Hogan B (1996) “Enhancer analysis of the mouse HNF-3 beta gene: regulatory elements for node/notochord and floor plate are independent and consist of multiple sub-elements”, Genes Cells, 1, 59-72

Thomas P & Beddington R (1996) “Anterior primitive endoderm may be responsible for patterning the anterior neural plate in the mouse embryo”, Current Biology, 6, 1487-1496

Thomas PQ, *et al*., (1998) “Hex: a homeobox gene revealing peri-implantation asymmetry in the mouse embryo and an early transient marker of endothelial cell precursors”, Development, 125, 85-94

Waldrip *et al*., (1998) “Smad2 signalling in extraembryonic tissues determines anterior-posterior polarity of the early mouse embryo”, Cell, 92, 797-808

Supplemental File 1: S1 dataset - Genes altered in *Lhx1* null DE cultures at day 5 and day 6 of differentiation

Supplemental File 2: S2 dataset - List of all proteins identified by MS from SF-epitope tagged Lhx1 samples, including output from the CPFP

Supplemental File 3: S3 dataset - Annotation of Lhx1 peaks identified by ChIP-seq. Dataset 3a contains overlapping peaks, dataset 3b shows all Lhx1 ChIP peaks identified

Costello_SupplFigS1

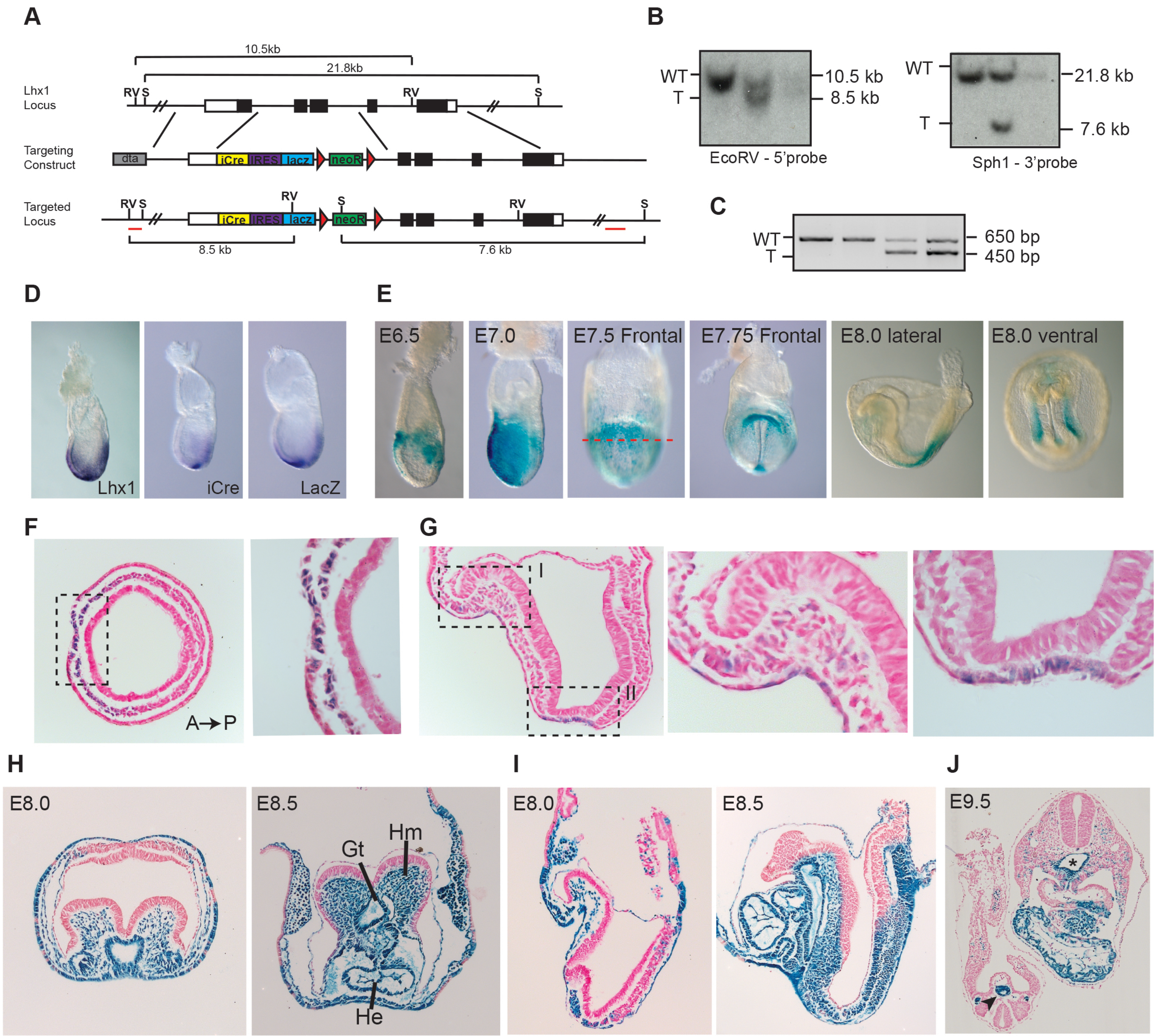

Costello_SupplFigS2

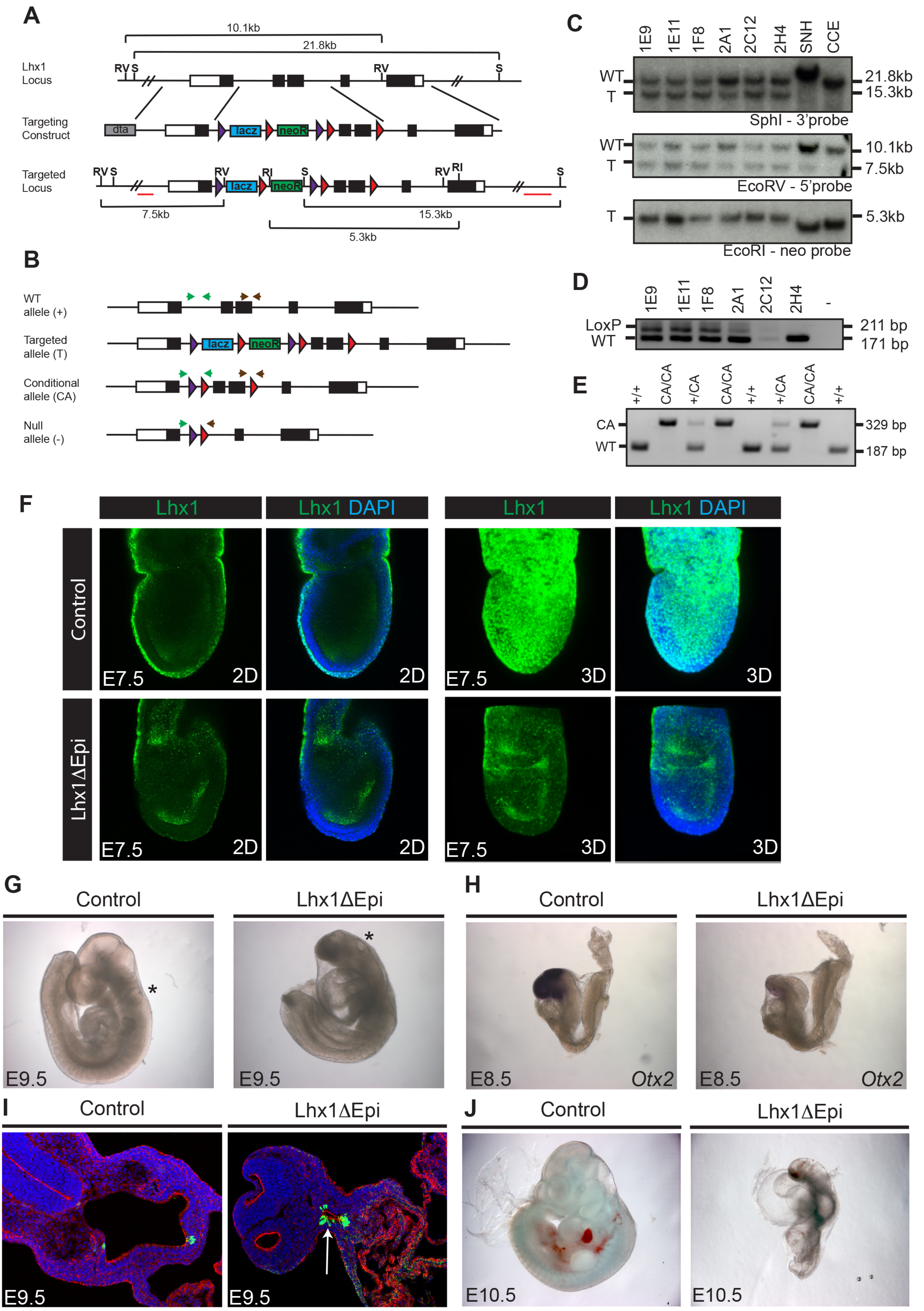

Costello_SupplFigS3

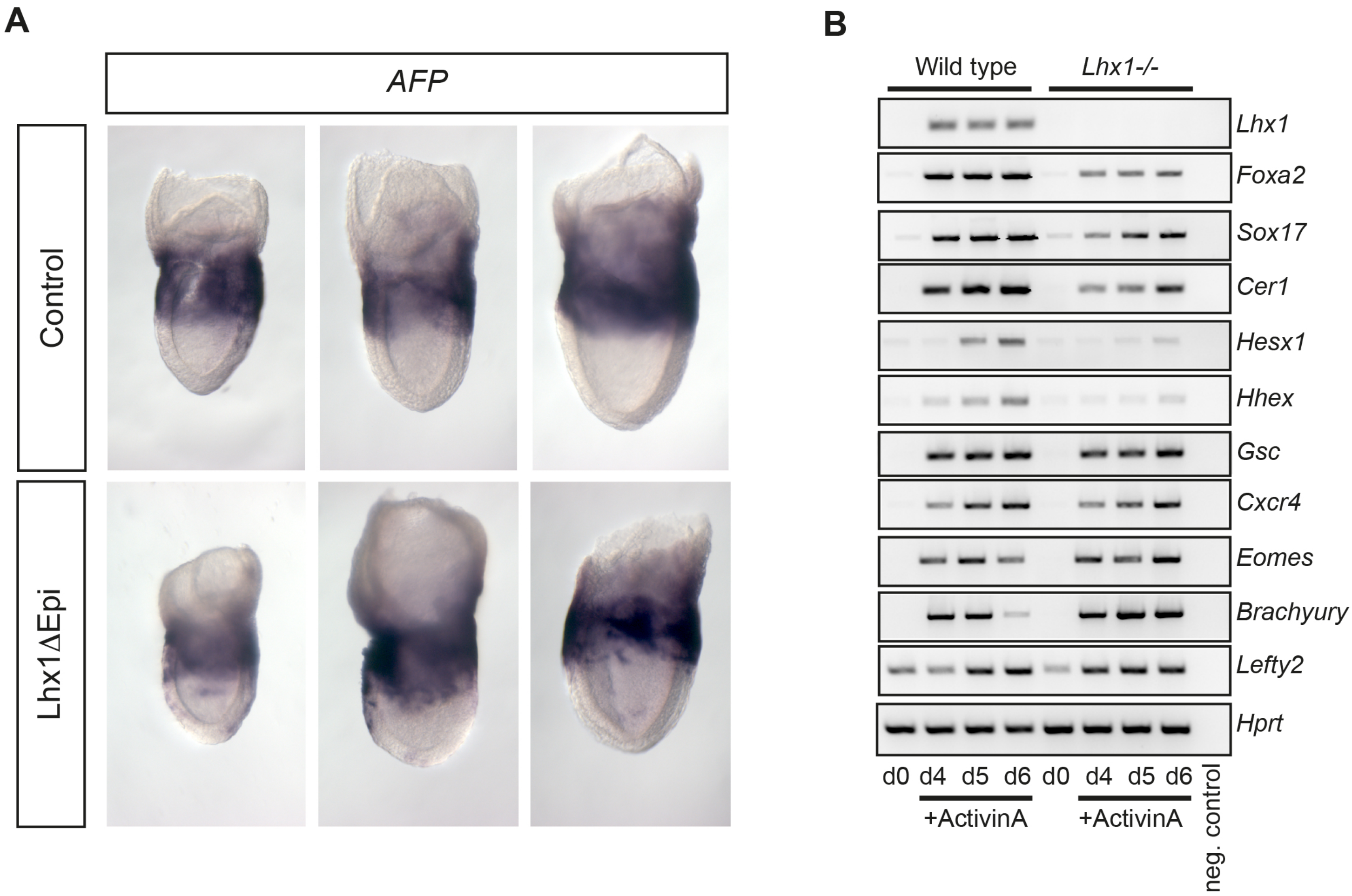

Costello_SupplFigS4

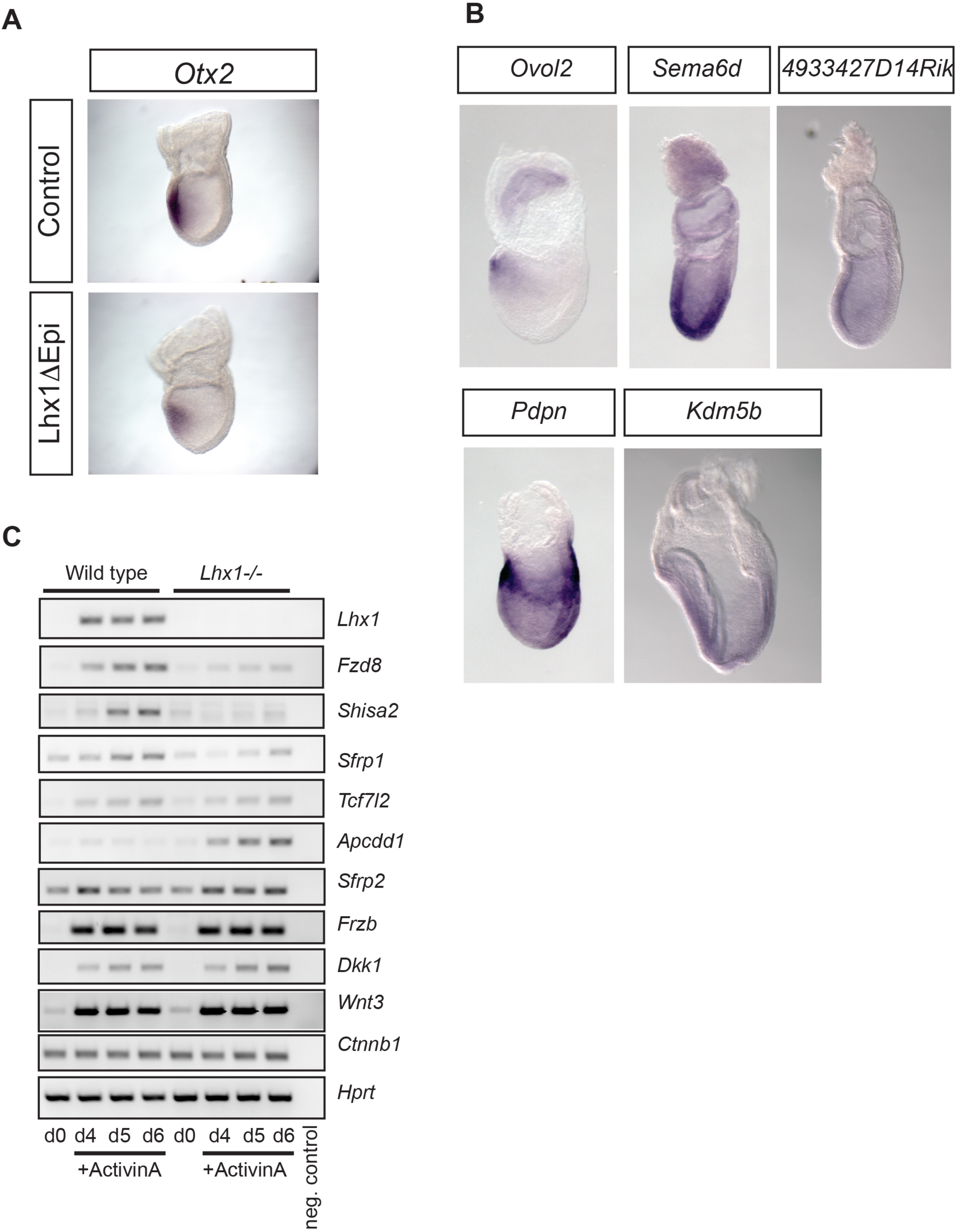

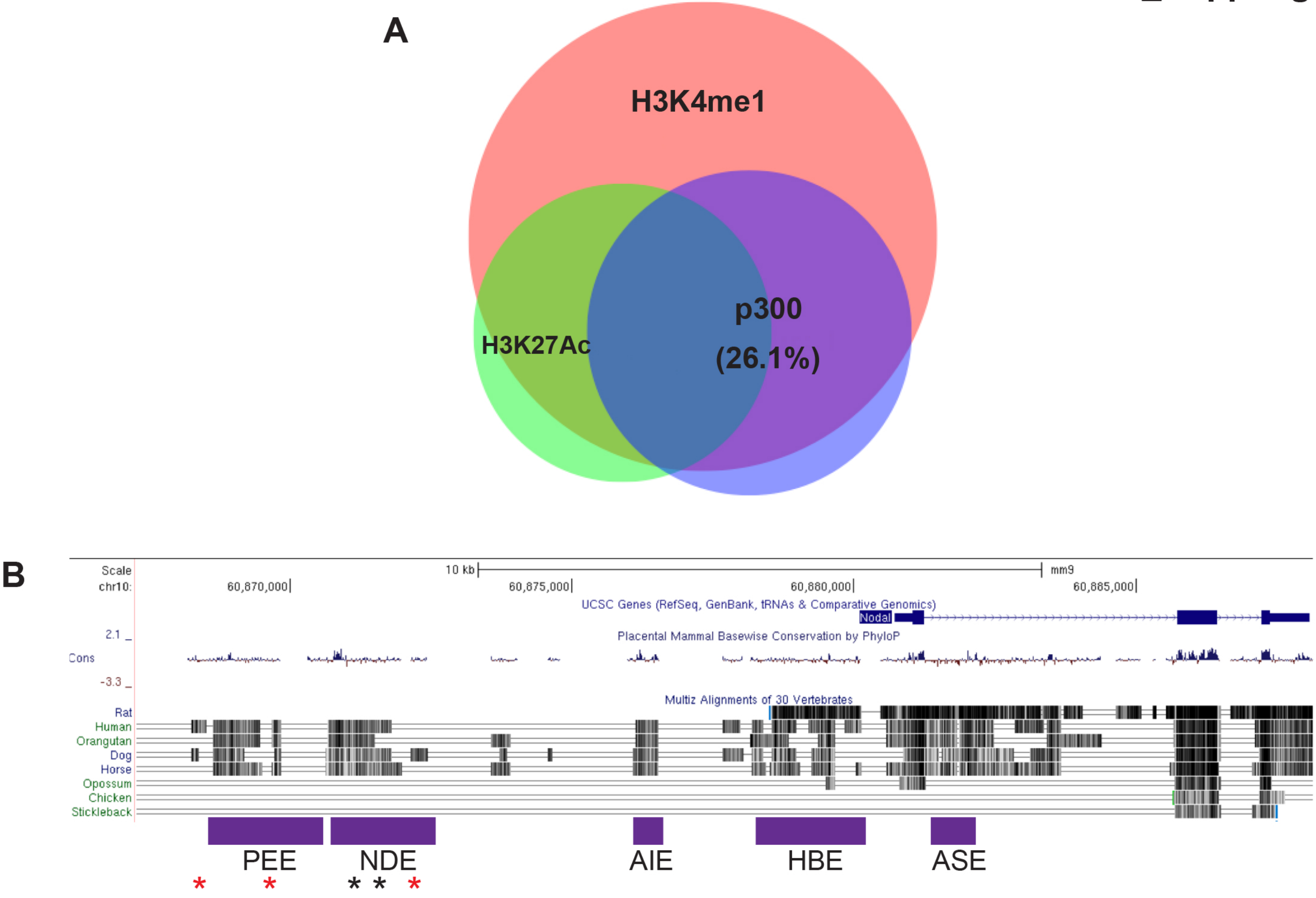

C

CACAGAGGTCTGCTTGCTCTGCCTCCTGAGTGCTGGGATCACAGAAGTTAGCTAGTGCCTAACCACCACACCCA
GTTTTTCTTTTAGTACCAACGTGACCTTGGACAGGAGCTGAAGCGGTGAGATCTTCAGTGTTTTCACCTGTGAAA
TGGGAATAAGTATGCCTCCTGAGAACAACTGGAAAGTCGCCCCCTTGAAGCTTCCTGTCACCGGTGCTGGGATG
ACCTGGAGCTGATGCAGCTGGCTCTGTTGTGTTGTCATTTGGGAGAATTTCACTCTGACGCAAGTGTCGTGTAC
ATCTGACATGGAATTGATACAGTGGCCTTCCTTCATTGTTTTATGAGAGTTCCACTCATTCATGTGTATTTAACTTA
GCCTGGAGCTCACTATGTAGACCGGGCTGGCCTTGAACTCACAGAGATCCTCCTGCCTCTGCCTCCTGAGCTCT
GGGTTGAAAGGTGAGCGTCACAACAGTCACAACAGCTGGCTTCTCTCATGCTTTTCTTCAGTCTTGCTTGGAAAG
CACACAAGTTTGGCACAGCCCCATCTCACCTCAATCCCCAGGTATCTAGAAGCAGATACTAGCCATAAGAATAAA
GGACTCCTAGCTTGGCTGGGGTGAGAACAGATTTTCACTTCCAAGGAATTAGCTGTTCTCTAAAGTACTTCCTGC
CCTGTTGAGAGTCCCTGGCTAATCGCATTAGCTGTCGGGAGCATTCTCAGTATGCGCACCTCTCCCGAGACACC
TTTGAAGTCCTGGGTCCTTTGATGTGAGTGGGGAATGCGGTTTGCGGGGAGATCCTGAGACCCCTGTGGTCAG
TGGCCCATAGAGCATGGGTGCTTGCTGCGAGTCTGAGCTGCAGGTGCCGCCTCCCTGCTTGCGGTGACGCAG
GGTGGGCTTTAGAGTCAAAGAGCTGAAAGCTAGTGCCAATCTTGCCATTAACCATTGTGACCTTCGTTCAGTCAC
CCAGCCTCTGAGCCTCCCATGCTTCCCCTTCAAATGAGGATTATAGAAACACCTCGTGAGGGGTGGGGGCGGG
GCAGTCCCTCATCTCACCTGCTCAAGAGCAAGACTCTCTGAAAAGACAAATCTTCCTGGGTCCCCTGCAGCCCT
GAAATGAAACTGCCTTCCAAAGACCTTAGAGAATTGTAAGCTTTATTCAAAAATATTCTCTTAACCGTGTTTTTCTC
TCAGATTTATAAACAAACATATGAAGAACATCATTATATATTTCCATTTTTGACACTTAGTTTTAGGAACATATTTGT
TCAGTCTTCTCTGTTTGTTTGTTTTTTGTTTGTTTGTTTGTTTTTTAGTTTTGCTTTTGAGAGCCCTGGATGTCCCG
AAACGTGCTATGTAAACCAGGCTGCCTTTGAACTTGTTGCGAATCCCTGCCTCTTTCTCTCAAACACTGGAATTA
CAGGCTGCTTGCTGGGAACCAAACCTGAGACTTCTGGAAGAGCAGCCAGTGCTCTAAACCACGGAGTCATCTC
CCCAGCTCCCAAAAATGCAATCTTTTAAAATTTTTTCAGTAAAGCATTACAGAATGGCTATGTAAGTCTTTACTGTT
TATACTTTTGGAATTATTAAAGATCCAAGGAACACTTAATAAAAGTAAAAAGTATCCCACAACTCTGACTGTGTGT
ACAGTTGTCAGTTTTTTTGTTTTGGCAACGTCTCTCAGTGTACAGCTGGTATACAGCTTTAACTGGACATGAAGG
CACATGTCTTTAATCCCAGCACTATGGCAGGCAGAGCAAGTGGATCTCTGTGAGGGTAGCATGGTCTACATAGT
AAGTTCCAGAACAGTCAGGCTACATAGTGAAACCCTGTCTCAATCAATCGATTGATCGATCGATCAATAATCAAT
CAATCAAGAACACAGTCTCTCTGGGCAGTGGTGGCGCACGCCTTTAATCCCAGCACTCGGGAGGCAGAGGCAG
GCAGATTTCTGAGTTCGAGGCCAGCCTGGTCTATAAAGTGAGTGCCAGGACAGCCAGGGCTACACAGAGAAAC
CCTGCCTCATAAAACCAAAAAAAAAAAAAAAAACCAAAAAACAAAAAAAACAAAAAACAAAACCACAGTCTCACAG
AGTCTGACTGGTCTGAGCCCCCTACTTGGTACAGACCAGGCTTGAACTCATGACCATCCTCCTGCCTCAACCTC
CTGAGTGCTGAGATTGTAGGCTCAAGCCACCAGAGAGCTCTGTCTTTTCTGTTTCTGTCTGTTTTGGGGTTTGGG
TTCTGAGATAGGTCTTGTGTGGCTCGGGCTAGCTTCTCACTAGTACTTACTAAGTAGTGAAGGATGCTTTTCAAC
TCCTGATCCCCCAGCCTCCTCTTTGAAGTGCTGGTAGCCAGATGCTTGCTTTTGCTCTTAGTAGGTACCTGACCT
GATCATTATCATATAGCAATGTGGGAGGACAGTGAGCACCTTTGCTCCCAGAAAACAGATCTGCTGTCATTATAT
CCCCCTACACAGACACACAGACATGGGGGGGGGGGCTCTTTTTAACCCAGGGCTCAGAGTTGAATCAGCACCT
CACCTTCAGCCCTGTGACTTCCTCCAAGGGACAAGGGAGAGGAGGGAGGCAGCAGATAGAGGCTGTGGGAAT
CGCAGAAACTCCACAGTGGGAGGAGAAGCCATCTTCCTGGTGGCCTCTGTGTTTGTTTCACAAAACCTTCCCAC
TCCCCGGGGTTCCCAGGCCAAGGGAGCCTTTCCCTCCTGACCCTGTGGGAAGGAAGCACCTGGCTGGGCTCC
TGCAGGACAGAGCCCAGGATGAGGAGTCTCAGGAACTTTAGATCTGCCTGGCCCCTGGCGCTAGACCGGCTCA
ATCCGGTCTGTCAGGCCAAGATGGTGCTAGGCCACCTCAGGACACGGCTCCCATGAGGCTCCTGAGAGGTTCA
CATAGAAGGACATGTAGGGGCCATGAGTCTGAATACTGGTTCATCATGACAGACGTCCGTCCTCTGGTCCTCTG
TCATGTTCCTAGAAGAGTTAGGGAGAAGACTCAGACTAGAGGAAAGCCAGGAAAGCCATGACCTGGGCTCCTGT
CCTTTTGGCAACCACAGCACTGCGAAGCATTTGCCCTGACTTCATCCCTTCCCACCTGTGTCCCCACCCCTACA
CCAGTGCAAGCACTGGGAAACTCATGCAACAAACAGGTCCTGTTTCCTTCTGTGTGTCAGAGATAGGCCAGTGG
CCCACTGATGAATTTGTGCTATAGCTCAAGGATGGGGTTGGGGACTGAGGAGGGGACAGTTAAAAATGAGCAAA
TGGTCACAAACACAATGAGTTCATCTCTGCTGTGGAGGGATGTGCCCTGGAGTTGCACAAGCATCTACCACAGA
GCAGTGGCACACAGGAGGCTCTTCAGCAGCAGCCAGCAGATGAGGGAAGGCCCCCCTGGGGCTGGGGGGGG
TGGCGAGCCCTGGCCTGGGTTTACAAGACAGGAGAACTTGGGGCCTATCCTAGCATGACTGCCTGTTAGAAACT
CTTCCCTAAGTGAGCGGCCAAACCTTCTGAGCCTTGATTTCTCTGTCTGTAATTCCGAAGAAGTCAACATCCTAT
CAGTTCCTAAGACTAATGAGGGGTCCACACTTAGCCTGCTTCCAGTTTACCCAACCAGGGCCATGTACACCAGT
CTAGTTGATCTGATCATGGGTGAGAGTCACCATAGGGGACAGTTAGG

PEE element
NDE element
Previously identified Rbpj motif
New identified Rbpj motif

Costello_SupplFigS6

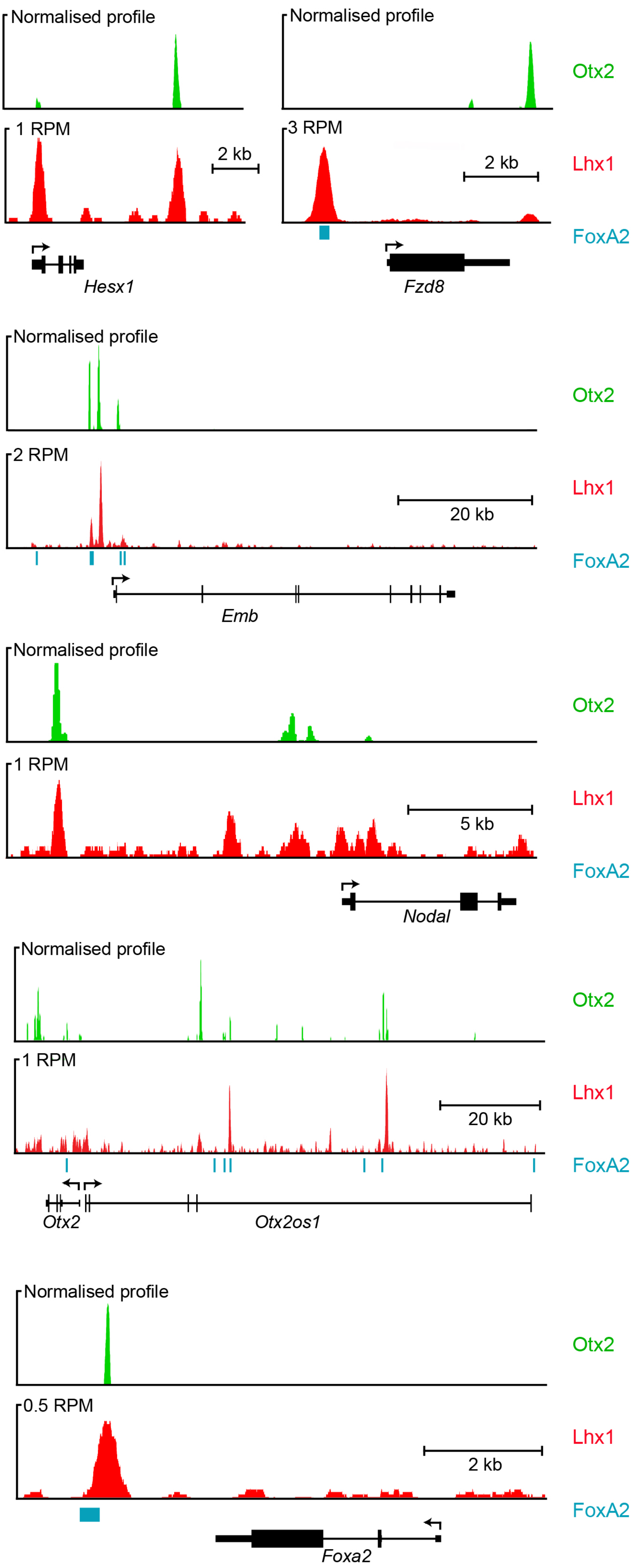

Supplement: Supplemental Material [file supp_29.20.2108_SuppMaterial.pdf]
